# Protein Disulfide Isomerase Family A Member 3 Knockout Abrogate Effects of Vitamin D on Cellular Respiration and Glycolysis in Squamous Cell Carcinoma

**DOI:** 10.3390/nu15214529

**Published:** 2023-10-25

**Authors:** Joanna I. Nowak, Anna M. Olszewska, Oliwia Król, Michał A. Żmijewski

**Affiliations:** 1 Department of Histology, Medical University of Gdansk, 1a Dębinki, 80-211 Gdansk, Poland; j.chorzepa@gumed.edu.pl (J.I.N.); anna.olszewska@gumed.edu.pl (A.M.O.); 2 Department of Biochemistry, Medical University of Gdansk, 80-211 Gdansk, Poland; oliwia.krol@gumed.edu.pl

**Keywords:** PDIA3, squamous cell carcinoma, mitochondria bioenergetic, vitamin D

## Abstract

PDIA3 is an endoplasmic reticulum disulfide isomerase, which is involved in the folding and trafficking of newly synthesized proteins. PDIA3 was also described as an alternative receptor for the active form of vitamin D (1,25(OH)_2_D_3_). Here, we investigated an impact of PDIA3 in mitochondrial morphology and bioenergetics in squamous cell carcinoma line A431 treated with 1,25(OH)_2_D_3_. It was observed that PDIA3 deletion resulted in changes in the morphology of mitochondria including a decrease in the percentage of mitochondrial section area, maximal diameter, and perimeter. The 1,25(OH)_2_D_3_ treatment of A431∆*PDIA3* cells partially reversed the effect of *PDIA3* deletion increasing aforementioned parameters; meanwhile, in A431WT cells, only an increase in mitochondrial section area was observed. Moreover, *PDIA3* knockout affected mitochondrial bioenergetics and modulated STAT3 signaling. Oxygen consumption rate (OCR) was significantly increased, with no visible effect of 1,25(OH)_2_D_3_ treatment in A431∆*PDIA3* cells. In the case of Extracellular Acidification Rate (ECAR), an increase was observed for glycolysis and glycolytic capacity parameters in the case of non-treated A431WT cells versus A431∆*PDIA3* cells. The 1,25(OH)_2_D_3_ treatment had no significant effect on glycolytic parameters. Taken together, the presented results suggest that PDIA3 is strongly involved in the regulation of mitochondrial bioenergetics in cancerous cells and modulation of its response to 1,25(OH)_2_D_3,_ possibly through STAT3.

## 1. Introduction

Protein disulfide isomerases are key oxidoreductase enzymes that play a role in the proper folding and assembling of proteins and their complexes [1]. An oxidoreductase family member, PDIA3 protein, has a broad range of functions from promoting protein folding in ER [2,3], participating in signal transduction through STAT3 in the nucleus [4,5], to pro-apoptotic activities in mitochondria [6]. Moreover, it was shown that PDIA3 can be localized in the mitochondria-associated membranes (MAMs) region of the endoplasmic reticulum closely associated with mitochondria [7,8]. Several studies have shown that PDIA3 functions as a chaperone to STAT3 protein and can modulate its transcriptional activity by regulating phosphorylation at the Y705 site [4,5,9,10]. On the other hand, phosphorylation of STAT3 at S727 residue alone targets the import of this transcription factor into mitochondria [11]. Moreover, it was suggested, that PDIA3 can suppress mitochondrial bioenergetic functions by inhibiting phosphorylation of the S727 site [12]. PDIA3 has been also linked to various diseases from neurodegenerative to cancer [13]. It was postulated that PDIA3 can be treated as a chemoprevention target and prognostic marker in cancer patients [14,15].

An active form of vitamin D, 1,25(OH)_2_D_3_, is a steroid hormone that regulates calcium–phosphorus homeostasis along with various cellular processes [16,17]. Canonically, vitamin D acts through the complex of its receptors: VDR and RXR, regulating the expression of hundreds of genes in the human genome [18,19]. However, not all effects of 1,25(OH)_2_D_3_ can be related to the genomic action of VDR–RXR heterodimer [20,21]. Consequently, PDIA3 was identified as a membrane-bound receptor for the active form of vitamin D (1,25D_3_-MARRS), responsible for non-genomic responses to the hormone [22,23,24]. It was shown that PDIA3 can form a complex with caveolin-1 and subsequently activated phospholipase A2-activating protein (PLAA) [25,26]. Thus, leading to the rapid action of 1,25(OH)_2_D_3_ via PKC [27]. Our recent studies have shown that genomic activity of 1,25(OH)_2_D_3_ strictly depends on VDR and only partially on RXRα [28], while deletion of *PDIA3* significantly modules the response [29]. Moreover, it was postulated that VDR can regulate the transcription of mitochondrial genes and directly interact with mitochondrial DNA [30]. However, several studies have shown the direct effects of 1,25(OH)_2_D_3_ on ion transport [31,32], including activity of mitochondrial membrane potassium channels [33]. Finally, pre-incubation with 1,25(OH)_2_D_3_ significantly deepened the effect of anti-cancer drugs on the mitochondrial respiration of patient-derived melanoma cells [34].

In our previous study, we established that PDIA3 is involved in 1,25(OH)_2_D_3_ action in the manner of gene expression profile and range of phenotypic effects, such as proliferation or migration [29]. Here, the impact of *PDIA3* deletion on mitochondrial morphology and bioenergetics in squamous cell carcinoma (A431) and its potential role in the action of vitamin D on mitochondria were investigated for the first time.

## 2. Materials and Methods

### 2.1. The 1,25(OH)_2_D_3_

The 1,25(OH)_2_D_3_ was purchased from Sigma-Aldrich (St. Louis, MO, USA). Stock solutions of 1,25(OH)_2_D_3_ were dissolved in ethanol and stored at −20 °C. At 100 nM concentration, 1,25(OH)_2_D_3_ was used in all experiments (the concentration of solvent (ethanol) was <0.05%).

### 2.2. Cell Cultures

Immortalized human basal cell carcinoma cell line (A431) was obtained from Synthego Corporation (Menlo Park, CA, USA). *PDIA3* knockout cell line was obtained with CRISPR/Cas9 technology as previously described [29]. The early passages 6 to 15 (after clonal selection) were used and deletion of PDIA3 was routinely confirmed via Western blot. Cells were cultured in DMEM high glucose medium (4.5 g/L) with the addition of 10% FBS, penicillin (10,000 units/mL), and streptomycin (10 mg/mL) (Sigma-Aldrich; Merck KGaA). Cell cultures were performed in the incubator with 5% CO_2_ at 37 °C. Before treatment with 1,25(OH)_2_D_3_, medium was changed to DMEM with 2% charcoal-stripped FBS.

### 2.3. Transmission Electron Microscopy (TEM)

The A431∆*PDIA3* cells were seeded onto a Petri dish (10 cm; VWR, Gdansk, Poland) at a density of 1 × 10^6^ cells/plate standard medium and after 24 h treated with 100 nM 1,25(OH)_2_D_3_. Consequently, the cells were fixed in 2.5% glutaraldehyde in 0.1 mM sodium-cacodylate buffer, scratched, and centrifuged. The cell pellets were then postfixed in 2% osmium tetroxide, dehydrated in ethanol, and infiltrated with a mixture of propylene. The pelleted cells were subsequently embedded to polymerize. Ultrathin sections (70 nm) were cut and, after dehydration, stained with uranyl acetate (Plano GmbH, Wetzlar, Germany) and lead citrate (Electron Microscopy Sciences, Hatfield, PA, USA). Samples were analyzed with an electron microscope (JEOL JEM-1200 EXII, University Park, PA, USA) at an acceleration voltage of 80 kV. Mitochondria from EM photos were counted in cellSens Olympus Software v 4.1. 

### 2.4. Seahorse Analysis

The effects of 1,25(OH)_2_D_3_ on the mitochondrial function of A431 ∆*PDIA3* were measured using the Seahorse Mito Stress Test following the manufacturer’s protocol. Briefly, 2 × 10^4^ cells/well were seeded on a Seahorse plate and after 24 h treated with 100 nM 1,25(OH)_2_D_3_ for 24 h. All essential compounds were diluted to final concentrations of 1 μM for Oligomycin, 1 μM for Carbonyl cyanide 4-(trifluoromethoxy) phenylhydrazone (FCCP), and 1 μM for Antimycin A/Rotenone, and cells were prepared according to Seahorse protocols. The experiment was run with Seahorse XF24 (Agilent Technologies, Santa Clara, CA, USA). After the Seahorse analysis, the cells were lysed with modified RIPA buffer supplemented with Roche (Basel, Switzerland) protease and phosphatase inhibitors cocktail (Roche, Basel, Switzerland), and protein concentration was measured with bicinchoninic acid assay (Thermo Fisher Scientific, Waltham, MA, USA) for data normalization. Each experiment was repeated at least three times, independently. The data were analyzed with Wave software version 1.1.1.3 (Agilent Technologies, Santa Clara, CA, USA), and the Student’s *t*-test was used to compare the mean fluorescence values between different experimental conditions. Basal respiration was calculated after subtraction of non-mitochondrial respiration (remaining OCR after Antimycin A addition). ATP-linked OCR was derived as the difference between basal and Antimycin A-inhibited OCR. Proton leak was calculated as the difference between OCR following Oligomycin A inhibition and OCR following Antimycin A inhibition. Maximal respiration was measured following the addition of FCCP. Spare capacity was calculated based on the difference between basal respiration and maximal respiration.

### 2.5. Fluorescent Probes

For fluorometric measurements, cells were seeded in 8-well chambers (MoBiTec Molecular Biology, Goettingen, Germany) at a density of 200.000 cells/well and incubated overnight (37 °C, 5% CO_2_). The next day, the medium was removed and cells were incubated with diluted to a final concentration of 2 µM JC-1 (Thermo Fisher Scientific, Waltham, MA, USA) or 100 nM MitoGreen (Thermo Fisher Scientific, Waltham, MA, USA) probes for 20 min. Then, solution containing fluorescent probe was replaced with 100 nM 1,25(OH)_2_D_3_ medium solution, and cells were grown for 24 h with live imaging under a microscope Olympus cell Vivo IX83 (Tokyo, Japan). For JC-1 (Thermo Fisher Scientific, Waltham, MA, USA) the ratio of red/green fluorescence intensity was analyzed with cellSens Olympus software version 4.1. For MitoGreen calculation, fluorescence intensity measurements were normalized against cell numbers before being expressed as percentages of control values.

### 2.6. Western Blotting

A431-derived cell lines were treated with 100 nM 1,25(OH)_2_D_3_ for 4, 8, and 24 h. The medium was removed from the plate, and cells were washed twice with PBS and were scratched from the plate. The solution was moved to an Eppendorf tube and centrifuged at 16,000× *g* for 10 min. The received cell sediment was dissolved in 100 µL of RIPA buffer (Thermo Fisher, Waltham, MA, USA). Concentration was determined by a modified Bradford Assay. For SDS-PAGE electrophoresis, 10% bottom gel and 5% upper gel were used. An equal amount of protein (20 µg) was loaded into each well. Electrophoresis was run at 90–110 V in the Bio-Rad apparatus. Proteins were transferred to PVDF membranes with the use of the Trans-Blot Turbo system (Bio-Rad, Hercules, CA, USA). After, the transfer membranes were blocked in 5% milk dissolved in TBS-T. The membranes were incubated with primary antibodies anti-STAT3 (Abclonal, Woburn, MA, USA), anti-pSTAT3 (Y705) (Abclonal, Woburn, MA, USA), or anti-pSTAT3 (S727) (Abclonal, Woburn, MA, USA), overnight at 4 °C. For loading control, membranes were stripped and re-probed with anti-β-actin antibodies (Abclonal, Woburn, MA, USA). Then, they were incubated with proper secondary fluorescent antibodies (AlexaFluor^®^ 790 or AlexaFluor^®^ 680 from Jackson ImmunoResearch, West Grove, PA, USA). Bands were visualized with Odyssey Clx system, and densitometry of bands was performed with Image Studio Software Version 5.2.

### 2.7. Immunofluorescence Staining

A431 cell lines were seeded in 8-well imaging chambers (MoBiTec Molecular Biology, Germany) at a density of 200.000 cells/well, incubated overnight (37 °C, 5% CO_2_). The next day, cells were treated with 1,25(OH)_2_D_3_ in DMEM medium supplemented with 2% charcoal-stripped FBS and 100 U/mL penicillin/streptomycin. After incubation time (4, 8, 24 h), cells were rinsed three times with PBS and fixed with 4% (*v*/*v*) formaldehyde solution, then washed three times with PBS, and permeabilized with 0.1% Triton X-100, blocked with 10% BSA in PBS for 1 h at RT and incubated with primary antibodies at 4 °C overnight (anti-STAT3, Abclonal, Woburn, MA 01801, United States). Following, the cells were rinsed three times with PBS, incubated with secondary antibodies for 1 h at RT (Alexa Fluor 488 anti-rabbit, Invitrogen, Waltham, MA, USA), then rinsed again with PBS, incubated with DAPI solution, and mounted with DAKO fluorescence mounting medium (S3025, Agilent Technologies, Santa Clara, CA, USA). The cells were visualized using fluorescence microscopy (Olympus Cell-Vivo IX 83, Japan) with camera ORCA-FLASH 4.0 and 60X objective (Hamamatsu, Shizuoka, Japan).

### 2.8. Bioinformatic Analysis

Transcriptomic data from a previous study were used to define mitochondrial genes expressed in A431∆*PDIA3* cells after 1,25(OH)_2_D_3_ treatment [29]. Venn analysis was performed with the online available tool [35].

### 2.9. Statistical Analysis

Statistical analysis was performed using GraphPad Prism version 7.05 (GraphPad Software, Inc., La Jolla, CA, USA). Data are presented as mean ± SD and were analyzed with a Student’s *t*-test (for two groups) or one-way ANOVA with appropriate post hoc tests (for more than two groups). Statistically significant differences are illustrated with asterisks: * *p* < 0.05, ** *p* < 0.01, *** *p* < 0.001, or **** *p* < 0.0001.

## 3. Results

### 3.1. Deletion of PDIA3 and 1,25(OH)_2_D_3_ Treatment Affect Morphology of Mitochondria 

**Figure 1:**
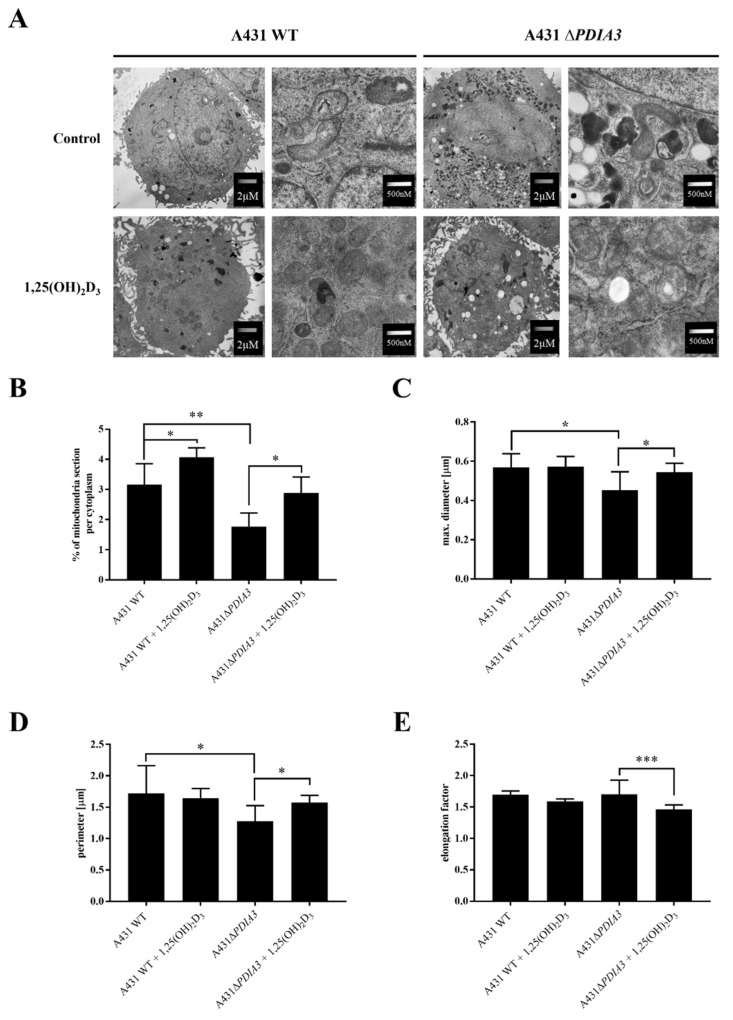
The 1,25(OH)_2_D_3_ treatment and PDIA3 deletion affect the morphology of mitochondria. (**A**) EM micrographs representing morphology of mitochondria of A431WT and A431∆*PDIA3* cells non-treated/treated with 1,25(OH)_2_D_3_ at two different magnifications. (**B**) Percentage of mitochondria section through the cytoplasm of A431WT and ∆*PDIA3* cells after 1,25(OH)_2_D_3_ treatment. Assessment of another mitochondrial parameter of A431 strains like (**C**) maximal diameter and (**D**) perimeter. (**E**) elongation factor. Data are expressed as mean ± SEM. * *p* < 0.05, ** *p* < 0.005, *** *p* < 0.001.

**Figure 2:**
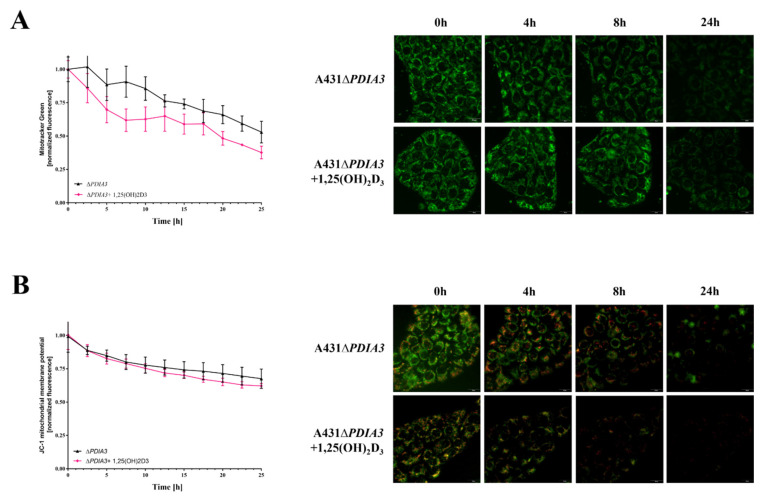
Mitochondrial surface area and membrane potential in *PDIA3* knockout A431 cell line after 1,25(OH)_2_D_3_ treatment. (**A**) The mitochondrial surface area in A431∆*PDIA3* cells stained with MitoTracker Green dye imaged with live microscopy Olympus cell Vivo IX83. (**B**) Mitochondrial membrane potential in A431 ∆*PDIA3* cells stained with JC-1 fluorescence probe with the use of live microscopy Olympus cell Vivo IX83.

The knockout of *PDIA3* in the A431 squamous cell carcinoma cell line was generated with the use of CRISPR/Cas9 technology as previously described [29]. The effects of *PDIA3* deletion and 1,25(OH)_2_D_3_ treatment on the morphology of mitochondria were investigated using transmission electron microscopy (TEM) (Figure 1A). A knockout of the *PDIA3* gene resulted in a twofold decrease in volume of mitochondria in comparison to wild type A431 (A431WT) cells, as shown by the percentage of the mitochondria section in whole cells observed using TEM. The treatment of A431WT or A431∆*PDIA3* with 1,25(OH)_2_D_3_ for 24 h resulted in a significant increase in the percentage of the mitochondria section (Figure 1B), but also in a reduction in the mitochondria diameter (Figure 1C) and perimeter (Figure 1D). The 1,25(OH)_2_D_3_ treatment of A431∆*PDIA3* cells partially reversed the effect of *PDIA3* deletion by increasing the aforementioned parameters, but there was no visible effect on A431WT cells. Interestingly, the elongation factor was not impaired by *PDIA3* deletion, but was decreased by 1,25(OH)_2_D_3_ treatment in the absence of PDIA3 (Figure 1E). Further investigation with use of fluorescence probes revealed that 1,25(OH)_2_D_3_ treatment noticeably affected mitochondrial surface area and mitochondrial membrane potential in A431∆*PDIA3* cells; however, the effect was not statistically significant (Figure 2A,B). 

### 3.2. PDIA3 Inhibits Mitochondrial Functions and Affects the Response to 1,25(OH)_2_D_3_ Treatment

**Figure 3:**
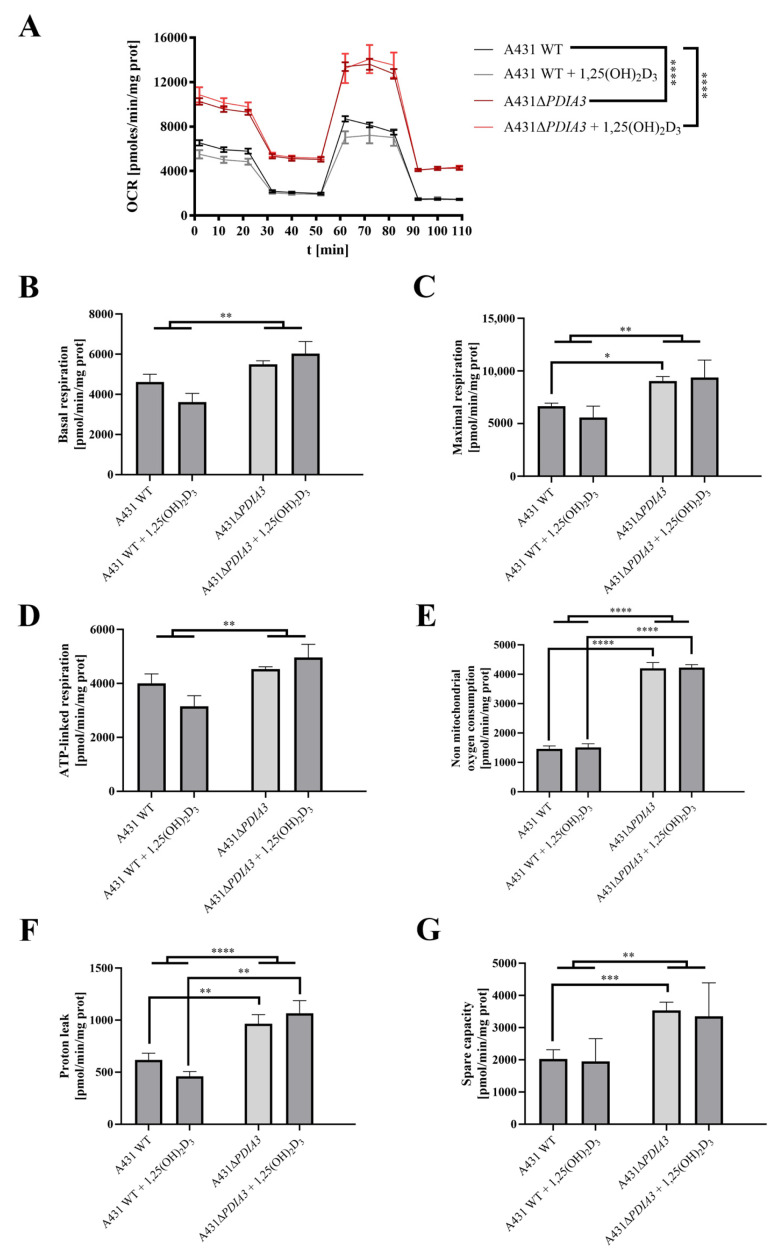
*PDIA3* deletion increases mitochondrial bioenergetics and abolishes the effect of 1,25(OH)_2_D_3_ treatment in A431 cells. (**A**) Representative traces of mitochondrial oxygen consumption rate of A431WT and A431∆*PDIA3* cells after 24 h of 1,25(OH)_2_D_3_ treatment. Mitochondrial respiration parameters: (**B**) basal respiration, (**C**) maximal respiration, (**D**) ATP-linked respiration, (**E**) non-mitochondrial oxygen consumption, (**F**) proton leak, and (**G**) spare capacity. Data are expressed as mean ± SEM. * *p* < 0.05, ** *p* < 0.005, *** *p* < 0.001, and **** *p* < 0.0001.

**Figure 4:**
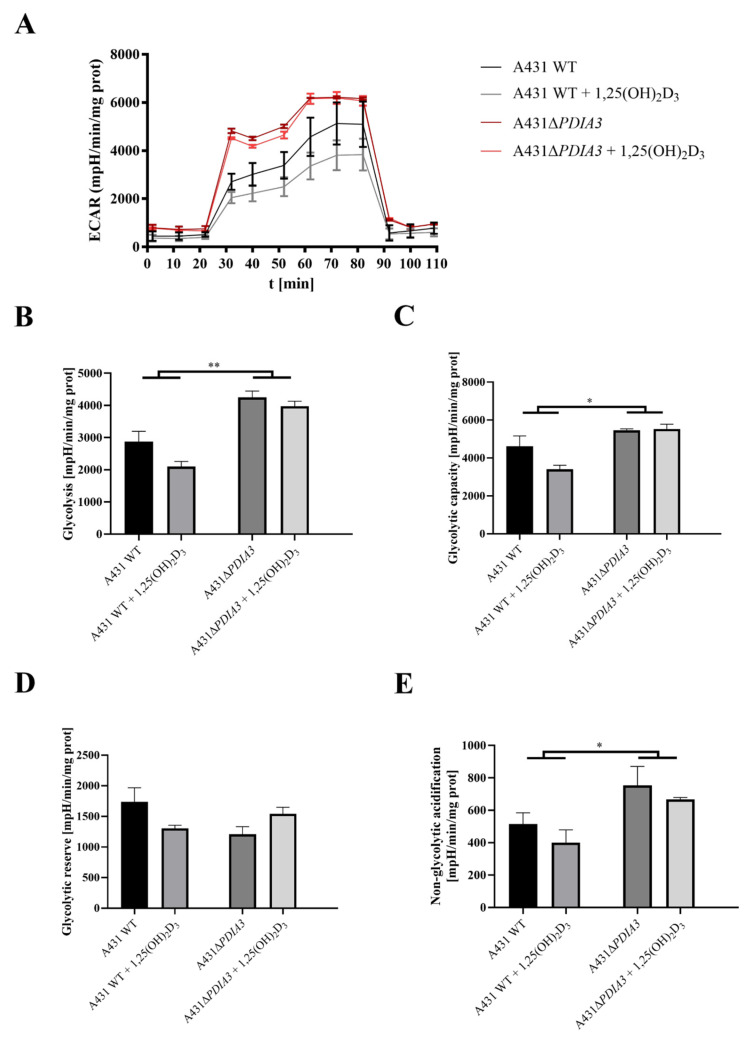
*PDIA3* deletion disrupts glycolytic functions and response to 1,25(OH)_2_D_3_ treatment of A431 strains. (**A**) Representative traces of mitochondrial Extracellular Acidification Rate of A431WT and A431∆*PDIA3* cells after 24 h of 1,25(OH)_2_D_3_ treatment. Mitochondrial glycolytic parameters: (**B**) glycolysis, (**C**) glycolytic capacity, (**D**) glycolytic reserve, and (**E**) non-glycolytic acidification. Data are expressed as mean ± SEM. * *p* < 0.05, ** *p* < 0.005.

The effect of 1,25(OH)_2_D_3_ treatment on mitochondrial bioenergetics in A431WT and A431∆*PDIA3* was determined using the Seahorse XF24. An oxygen consumption rate (OCR) was monitored in real-time with the following addition of Oligomycin, FCCP, Rotenone, and Antimycin. It was observed that in A431∆*PDIA3*, the OCR, expressed in pmoles/min/mg of protein, is significantly higher than in A431WT cells, and 1,25(OH)_2_D_3_ treatment did not affect those results (Figure 3A). Overall, it was shown that deletion of *PDIA3* enhances all parameters of oxidative phosphorylation; however, despite the clear trends, some results did not reach statistical significance. To increase the strength of comparison, data for treated and non-treated cells were combined, and the effect of PDIA3 on cellular bioenergetics was reanalyzed (Figure 3). In a case of basal respiration (Figure 3B) and ATP-linked respiration (Figure 3D), a statistically significant increase was observed after deletion of *PDIA3*, and for 1,25(OH)_2_D_3_ treated cells decrease in A431WT and increase in A431∆*PDIA3* was observed (Figure 3B). Further *PDIA3* deletion increased maximal respiration, but this parameter was not affected by 1,25(OH)_2_D_3_ treatment (Figure 3C). Interestingly, for non-mitochondrial oxygen consumption, a threefold increase in A431∆*PDIA3* cells was observed, with no further effect of 1,25(OH)_2_D_3_ (Figure 3E). Similarly, an increase in proton leakage was observed, but with adverse trends in A431WT and A431∆*PDIA3* cells after 1,25(OH)_2_D_3_ addition (decrease in A431WT and increase in A431∆*PDIA3*; Figure 3F). A mitochondrial spare capacity was increased twofold in A431∆*PDIA3* cells in comparison to wild-type cells. No effect of 1,25(OH)_2_D_3_ treatment on this parameter was observed (Figure 3G). Next, the impact of *PDIA3* knockout and/or 1,25(OH)_2_D_3_ treatment on glycolysis was investigated using glycolytic stress tests. The Extracellular Acidification Rate (ECAR) was measured in real-time by adding glucose to the medium on Seahorse XF24. Significant changes in the ECAR were observed between the 30th and 70th minute of the assay in the case of non-treated A431WT cells versus A431∆*PDIA3* cells (Figure 4A). Deletion of *PDIA3* gene enhanced levels of glycolysis and other parameters (Figure 4B,E), except for glycolytic capacity and reserve (Figure 4C,D). In general, treatment of A431WT cells with 1,25(OH)_2_D_3_ resulted in a decrease in glycolysis (Figure 4B), glycolytic capacity (Figure 4C), glycolytic reserve (Figure 4D), and non-glycolytic acidification (Figure 4E), but the results were marginally statistically significant. The tendency was not so pronounced in A431∆*PDIA3* cells.

### 3.3. PDIA3 Knockout Affects the Expression of Mitochondrial Genes 

**Figure 5:**
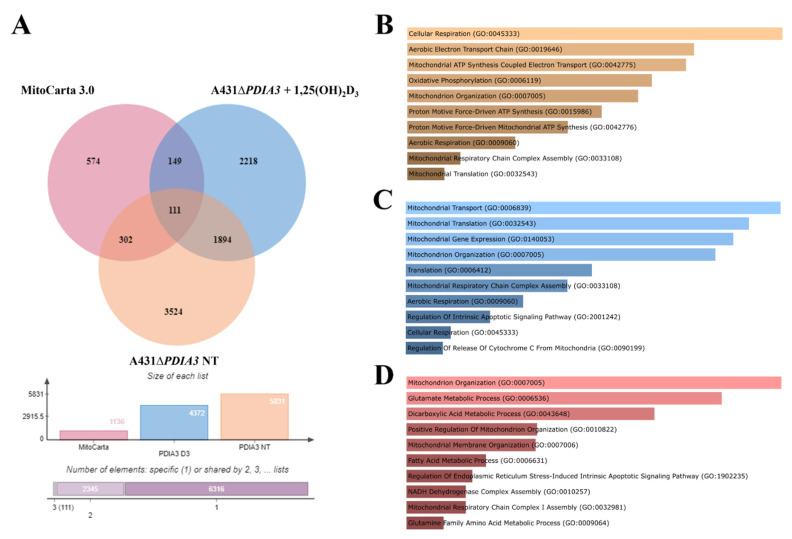
*PDIA3* deletion alters the expression of mitochondrial genes after 1,25(OH)_2_D_3_ treatment in A431 cells. (**A**) Comparison of mitochondrial genes from MitoCarta 3.0, A431WT, and A431∆*PDIA3* cells treated with 1,25(OH)_2_D_3_. Gene ontology of mtDEGs from A431∆*PDIA3* in terms of (**B**) biological process, (**C**) molecular functions and (**D**) cellular components.

In previous work, the effects of 24 h incubation with 1,25(OH)_2_D_3_ at 100 nM concentration on the transcriptome of A431∆*PDIA3* were studied [29]. To assess the impact of PDIA3 on the expression of the genes related to mitochondria, a previously obtained dataset of differentially expressed genes (DEGs; false discovery rate (FDR) = 0.05) from A431∆*PDIA3* non-treated and 1,25(OH)_2_D_3_-treated cells was used. The dataset was compared with mitochondria-associated genes (mtDEGs) from MitoCarta 3.0 database [36] via Venn analysis [35] (Supplementary Table S1), followed by gene ontology (GO) analysis [37]. The data are deposited in Sequence Read Archive (SRA) under accession number PRJNA926032. Venn analysis revealed 5831 DEGs expressed after *PDIA3* deletion in A431 cells and 4372 DEGs after treatment of A431∆*PDIA3* cells with 1,25(OH)_2_D_3_. Among those, 302 mtDEGs identified in A431∆*PDIA3* were affected solely by *PDIA3* deletion, while 149 mtDEGs were changed by 1,25(OH)_2_D_3_ treatment (Figure 5A). Interestingly, 111 mtDEGs were commonly regulated after *PDIA3* deletion and 1,25(OH)_2_D_3_ treatment. GO analysis of molecular processes revealed that deletion of *PDIA3* in A431 cells alone mainly affected cellular respiration (GO:0045333), aerobic electron transport chain (GO:0019646), and mitochondrial ATP synthesis (GO:0042775) (Figure 5B). Curiously, the 1,25(OH)_2_D_3_ treatment of knockout cells changed entirely different molecular processes linked to mitochondrial transcription/translation, such as mitochondrial translation (GO:0032543), mitochondrial gene expression (GO:0140053), and mitochondrial transport (GO:0006839) (Figure 5C). mtDEGs affected by both deletion of *PDIA3* and 1,25(OH)_2_D_3_ treatment were connected with mitochondrion organization (GO:0007005), glutamate (GO:0006536), and dicarboxylic acid (GO:0043648) metabolic processes (Figure 5D). 

### 3.4. PDIA3 or VDR Deletion Disrupts STAT3 Signaling Changing Response to 1,25(OH)_2_D_3_

**Figure 6:**
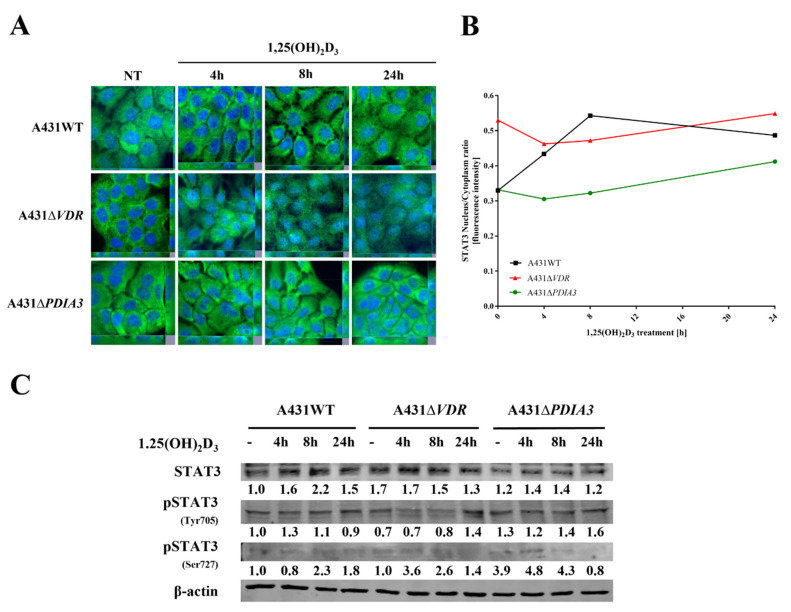
*PDIA3* deletion affects STAT3 signaling in A431 squamous cell carcinoma. (**A**) Fluorescence images of A431 cell lines treated with 1,25(OH)_2_D_3_ for 4, 8, or 24 h and stained with anti-STAT3 antibody and DAPI. (**B**) STAT3 nucleus/cytoplasm ratio in A431 sublines. (**C**) Analysis of protein levels of STAT3, pSTAT3 (Y705), and pSTAT3 (S727) in A431WT and *VDR* or *PDIA3*-deficient knockout cell lines.

As STAT3–PDIA3 interaction is widely described in the context of cell signaling, including regulation of cellular respiratory [4,12,38], it was checked whether 1,25(OH)_2_D_3_ can affect this signaling and, if so, how PDIA3 is involved in the process. To elucidate an impact of 1,25(OH)_2_D_3_ on STAT3 translocation into the nucleus, immunofluorescent staining was performed (Figure 6A). In the case of A431WT cells, we observed translocation of STAT3 into the nucleus after 1,25(OH)_2_D_3_ treatment, with the highest intensity ratio after 8 h of incubation. Deletion of the *VDR* (vitamin D receptor) decreased the basal signal, both nuclear and cytoplasm, resulting in a higher nucleus/cytoplasm ratio for STAT3, but the effect of 1,25(OH)_2_D_3_ treatment was not observed. Interestingly, deletion of PDIA3 did not change basal intensity for STAT3, but similarly to A431∆*VDR* cells, there was no visible effect of 1,25(OH)_2_D_3_ treatment (Figure 6B). Secondly, levels of STAT3 protein and its two phosphorylation sites (Ser727, Tyr705) were examined using Western blot analysis (Figure 6C). The amount of total STAT3 increased in time, with the highest level observed after 8 h of incubation of A431WT cells with 1,25(OH)_2_D_3_. The deletion of *VDR* increased the initial level of STAT3 and abrogated an increase induced by 1,25(OH)_2_D_3_ treatment. Similarly, *PDIA3* deletion slightly increased the basal amount of STAT3 with no effect from 1,25(OH)_2_D_3_ treatment. Finally, phosphorylation of STAT3 at the Y705 site occurred after 4 h of treatment solely in A431WT cells treated with 1,25(OH)_2_D_3_. Interestingly, the STAT3 phosphorylation at the S727 site, which is lined to mitochondria, was strongly increased by both knockouts in A431 cells (A431∆*VDR* and A431∆*PDIA3*) and further amplified by treatment with 1,25(OH)_2_D_3_ for 4 h. 

## 4. Discussion

PDIA3 is a pleiotropic member of the oxidoreductase enzyme family, which is involved in a broad range of cellular processes, including protein folding and assembly, through the formation and remodeling of disulfide bridges [39]. PDIA3 has been strongly associated with various types of cancer as a prognostic biomarker (primary ductal breast cancer, prostate cancer, glioblastoma) and its overexpression is associated with poor outcomes of patients [15,40,41,42]. Thus, this study focused on a squamous cell carcinoma cell line with deletion of *PDIA3* (A431∆*PDIA3*) as a model. In our previous study, we showed that deletion of *PDIA3* not only affects cellular physiology, but also plays an indispensable role in biological activities of 1,25(OH)_2_D_3_ including genomic response [29]. Previously, we observed that *PDIA3* deletion alone modulates expression of nearly 2000 genes, among which, 269 were 1,25(OH)_2_D_3_-regulated. Furthermore, *PDIA3* knockout changed the expression of 1,25(OH)_2_D_3_-dependent genes, suggesting its role as a modulator of genomic response. The present study aimed to assess the impact of PDIA3 on morphology and bioenergetics of mitochondria and its role in 1,25(OH)_2_D_3_ action on mitochondria in squamous cell carcinoma A431 cell line. To our knowledge, this is the first study investigating the role of PDIA3 in the mitochondrial activity of 1,25(OH)_2_D_3_. Hence, we are presenting data indicating that the deletion of *PDIA3* affects the morphology of the A431 cells, especially mitochondria. Knockout of *PDIA3* led to the decrease in total mitochondria surface and size within the cell, and 1,25(OH)_2_D_3_ treatment reversed the effect of deletion to some extent. Interestingly, after *PDIA3* deletion we did not observe any statistically significant change in mitochondrial potential, even though vitamin D analogues have previously been shown to abolish the effects of hydrogen peroxide on mitochondrial membrane potential in immortalized HaCaT keratinocytes, thus protecting mitochondria against oxidative damage, but the effect was time-dependent [43]. Furthermore, it seems that the effect on mitochondrial membrane potential might be cell-type dependent.

As the deletion of *PDIA3* was shown to affect cellular responses to 1,25(OH)_2_D_3_ treatment [29] and here we observed changes in the morphology of mitochondria, we decided to assess the impact of that deletion on mitochondria bioenergetics in A431 cells. All of the respiratory parameters of A431 cells were considerably elevated after *PDIA3* deletion. The presented data are in line with results published by Keasey et al., who showed that PDIA3 inhibits respiratory function in endothelial cells and *C. elegans* [12]. Previously, PDIA3 was localized within mitochondria, where it associates with mitochondrial μ-calpain, possibly playing a significant role in apoptotic signaling [44]. Moreover, PDIA3 was colocalized with STAT3 [45], suggesting its role in the modulation of STAT3 signaling within cells [46]. As those results suggested the possible involvement of PDIA3 in the modulation of 1,25(OH)_2_D_3_-induced STAT3 signaling, we analyzed levels of STAT3 protein together with its two phosphorylation sites at Tyr705 and Ser727. Our results suggest that VDR together with PDIA3 are necessary for the regulation of both phosphorylation sites via 1,25(OH)_2_D_3_. In our recent work [29], we identified the cyclooxygenase-2 coding gene (*PTGS2*) as a PDIA3-dependent gene. Interestingly, the expression of *PTGS2* is known to be regulated by STAT3 [47]. Consequently, we observed that *PDIA3* deletion abrogated the induction of the expression of *PTGS2* via 1,25(OH)_2_D_3_ [29]. Here, we are presenting results indicating impaired STAT3 phosphorylation at site Y705 in *PDIA3* or *VDR* knockouts, suggesting that both proteins are necessary for the regulation of nuclear STAT3 phosphorylation. The enhanced oxygen consumption rate after *PDIA3* deletion is further supported by increased phosphorylation of STAT3 at S727 residue in A431∆*PDIA3* cells. This observation is consistent with previous studies showing that PDIA3 can inhibit STAT3 phosphorylation and thereby influence mitochondrial bioenergetics [12]. Recently, Peron and coworkers showed that phosphorylation is needed for mito-STAT3 to exert its mitochondrial functions [48]. Here, for the first time, it was shown that phosphorylation of STAT3 at Tyr705 and Ser727 can be induced by 1,25(OH)_2_D_3_ and depend on the presence of both VDR and PDIA3. 

Interestingly, we observed that the lack of PDIA3 abrogated the effects of 1,25(OH)_2_D_3_ on energy production parameters, suggesting its involvement in cellular bioenergetics. Furthermore, an increase in glycolytic parameters was acknowledged after *PDIA3* deletion while 1,25(OH)_2_D_3_ treatment decreased glycolysis in wild-type A431 cells, but no effect was observed in A431∆*PDIA3*. This is in agreement with other studies showing reduced glycolysis after vitamin D treatment in breast cancer cells [49] and colorectal cancer [50].

Recently, we showed that *PDIA3* deletion alters the expression of more than 2000 genes and modulates genomic response to 1,25(OH)_2_D_3_ [29]. Here, we focused on genes related to mitochondria. However, we did not identify any PDIA3-dependent mtDEGs, which were also regulated by 1,25(OH)_2_D_3_, even though deletion of *PDIA3* alone changed the basal expression of mtDEGs, regulating different processes connected with cellular respiration. In a recent work [28], it was shown that 1,25(OH)_2_D_3_ affects differently morphology and bioenergetics of cancerous and non-cancerous cells through genomic pathways regulated by VDR and partially by RXRA [44]. However, it is clear that PDIA3 somehow modulates the response of cancerous cells to 1,25(OH)_2_D_3_ treatment in terms of mitochondrial morphology and bioenergetic; therefore, it supports our previous founding that PDIA3 possibly functions as a modulator of genomic response to 1,25(OH)_2_D_3_. Interestingly, Gezen-Ak and coworkers suggested that VDR affects directly mitochondrial DNA expression after 1,25(OH)_2_D_3_ treatment [30], opening new possibilities for the direct impact of 1,25(OH)_2_D_3_ and VDR on mitochondria; however, the presence of VDR in mitochondria is still under debate [20]. It was also postulated that VDR and PDIA3 are located at the cell membrane and are responsible for the trafficking of vitamin D and activation of fast membrane responses to this powerful secosteroid (see [20] for further discussion). However, the nature of VDR and PDIA3 interaction still remains to be solved.

Taken together, we have shown that *PDIA3* deletion affects mitochondria morphology and bioenergetics most likely through STAT3 regulation, as well as mitochondrial response to 1,25(OH)_2_D_3_. As we did not identify any PDIA3-dependent mtDEGs, we suggest that the main effects of 1,25(OH)_2_D_3_ are genomic actions mediated by VDR and partially by RXRA [28]. As PDIA3 was found also in mitochondria, the direct impact on mitochondrial structure and function cannot be excluded [12,44,51]. Data presented here broaden our knowledge about the role of PDIA3 in 1,25(OH)_2_D_3_ activities on mitochondria and open new perspectives to further explore topics of PDIA3-STAT3 regulation in 1,25(OH)_2_D_3_ action. A potential limitation of this study was the use of the A431 squamous cell carcinoma cell line, rather than primary keratinocytes; however, primary cell lines are not suitable for producing stable knockouts. Moreover, our previous studies showed that the effects of 1,25(OH)_2_D_3_ on cancer cell physiology, including mitochondrial function, were more pronounced in A431 cells compared to HaCaT keratinocytes. Most importantly, 1,25(OH)_2_D_3_ treatment at least partially reversed the expression of cancer-related genes [28,29] and modulated mitochondrial activity as shown here.

## Data Availability

The data presented in this study are available on request from the corresponding author.

## Supplementary Materials

The following supporting information can be downloaded at: https://www.mdpi.com/article/10.3390/nu15214529/s1, Table S1. Venn analysis of MitoCarta 3.0, A431∆*PDIA3* NT and A431∆*PDIA3* + 1,25(OH)_2_D_3_.

## Abbreviations

PDIA3	Protein Disulfide Isomerase Family A Member 3
OCR	Oxygen consumption rate
ECAR	Extracellular Acidification Rate
STAT3	Signal Transducer And Activator Of Transcription 3
VDR	Vitamin D receptor
RXRA	Retinoid X Receptor Alpha
PLAA	Phospholipase A2 Activating Protein
PKC	Protein Kinase C
TEM	Transmission electron microscopy
mtDEGs	mitochondria-associated differently expressed genes
GO	Gene ontology
