# Supplementary material for: Protein Disulfide Isomerase Family A Member 3 Knockout Abrogate Effects of Vitamin D on Cellular Respiration and Glycolysis in Squamous Cell Carcinoma
Source: Nutrients. 2023 Oct 25;15(21):4529. doi: 10.3390/nu15214529 (PMC10650882; doi:10.3390/nu15214529)
Supplement: Supplementary file 1 [file nutrients-15-04529-s001.zip › nutrients-2673051-supplementary.pdf]

MitoCarta	PDIA3 D3	PDIA3 NT	MitoCarta|PDIA3 D3	MitoCarta|PDIA3 NT	PDIA3 D3|PDIA3 NT	MitoCarta|PDIA3 D3|PDIA3 NT
COQ7	EFL1	PDIA3	COX5A	CYC1	SLC44A5	PDHA1
SDHA	ANO10	EPHA3	CS	SDHB	SLCO1A2	COQ9
COQ5	ARHGEF16	KRT81	DLAT	UQCRC1	SH3TC1	DBT
MRPL12	GASK1B	ZNF888	ATP5F1E	ATP5F1A	RNF39	LRPPRC
ATP5F1D	TMOD1	FAM174B	MRPS15	OGDH	SCEL	NDUFS1
ISCA2	IL1RL1	PDE3A	MRPL34	UQCRC2	BAIAP2L2	OPA1
PMPCB	SEMA3B	ZNF83	LETMD1	SDHD	CD86	ECHDC3
UQCRFS1	SP140L	CNTN1	AUH	MDH2	TIMP3	APOOL
PDHB	OLR1	IFNGR2	NDUFB8	MRPS27	ELL2	ACADM
MRPS35	SERINC2	CDH13	MRRF	GRPEL1	GOLM1	SLC25A4
UQCRQ	CLMN	ZNF468	MTIF2	DLST	PTPN1	ACO2
MRPL53	GPRC5A	BAG6	SURF1	PDHX	WNT7B	HIBCH
PDK4	DHRS3	RPS6KA2	GUF1	ATP5F1B	COL16A1	HSPA9
GFM1	DUSP10	PLTP	SDHAF2	SDHAF4	G6PD	MRPL40
MPC2	LBH	TMEM50B	MTCH2	UQCR10	ZNF185	SUCLG2
MRPL46	EAF2	CLCA2	HADH	COQ3	TMPRSS4	SLC25A1
SLC25A3	NET1	P2RY1	ACAT1	IDH3A	CASC9	NDUFA2
MRPS23	ARHGEF28	NEFL	MALSU1	TIMM10	ZNF704	ALDH6A1
FH	LINC00649	C12orf75	CLPX	UQCRH	PPP3CA	ALDH2
PMPCA	LDLRAP1	ABCC4	MECR	HIGD2A	KDM1A	SLC25A12
ISCA1	BBOX1-AS1	SSBP2	SLC25A11	ECHS1	AFAP1L2	DNAJA3
SUCLA2	GNAI2	GALNT14	TACO1	COX6A1	PKP1	NDUFB2
IARS2	APOBEC3B	HLA-DPA1	MRPL33	COX15	KLK6	COQ10B
COX11	MOK	RARRES1	SLC25A42	AFG3L2	CYP3A5	IDH2
ETFDH	NTN4	ZNF808	TOMM7	HADHA	JUNB	MRPL9
MRPL2	LINC01748	SERPINE2	GATB	ETFA	AIG1	NDUFA8
BCKDHA	KLK13	AJAP1	MRPL28	CPT2	ADGRD2	GLS
ATP5PO	PEX13	VPS52	NFU1	IDH3B	FAM126A	VWA8

NDUFS7	CALML3	MLLT3	ACADVL	LARS2	ADGRE5	AK4
BCKDHB	COPG2	FADS2	ABCB7	ACADS	ITGB6	TXNRD2
NDUFV2	HOMER3	RAB31	ALDH9A1	LETM1	DKK1	MTHFD1L
COQ6	WEE1	HLA-DQB1	MARS2	ATP5ME	FUT3	ATAD1
MRPL43	TSKU	GOLIM4	PPIF	SUCLG1	ABHD12	MTG2
ABHD11	HSD17B2	PFKM	L2HGDH	ATP5PF	MELTF	TIMM17B
LONP1	CETN2	KCNMA1	ETHE1	DLD	PDGFA	CARS2
MRPL10	CDA	LEMD2	MRPL20	AIFM1	ZNF641	ALDH1B1
NDUFS8	RAB38	RASSF8	ACAA2	TIMM9	AKR1C3	COX10
IMMT	MXD1	GRIK2	AFG1L	SAMM50	CAPNS1	AKAP1
FECH	CREG2	PON2	ATP5IF1	NDUFS2	ELAPOR2	MCUR1
PHB	DUSP5	PELI2	POLDIP2	NDUFV1	PLEKHG3	TMEM14C
MRPS2	CHST15	SLC4A2	SLC25A13	SUPV3L1	GEM	PDP1
TIMM13	BLVRA	TNIK	SLC25A19	PRDX3	ZBED6CL	PTCD2
MRPL16	MIR4713HG	CDK14	NDUFA10	GHITM	KCNK5	NAXE
IDH3G	ST6GALNAC1	ADAMTS15	MSRB2	LYRM4	PADI3	MTHFD2
SDHC	SGSM2	C1S	LACTB	NDUFB5	CD14	GSR
COQ10A	MACC1	KLHL13	ADCK5	MRPS18A	RNF145	GADD45GIP1
GATD3A	METAP1	MAPK14	THEM4	NDUFB9	FOCAD	ALDH18A1
TXN2	SULT1A1	EHMT2	MRPS10	NDUFA6	MFGE8	TMEM65
COX6C	ARSI	STC1	MRPL41	MICOS10	ERMP1	PRDX5
SLC25A20	CLCF1	ADGRL2	MRPS6	VDAC1	STEAP4	SLC25A46
MRPL1	RHOF	KLHDC2	MCCC2	CLPP	TENT5A	CROT
TIMM44	IRF1	ZNF76	MRPL51	NDUFS4	RAPGEF3	ALDH7A1
COX17	KCNN4	ALOX12	FAM210A	C1QBP	IGFBP3	YME1L1
SOD2	MVK	HLA-A	NIPSNAP2	PITRM1	DGAT2	ABCD3
COX6B1	CLCA4	PRKCQ	NDUFB7	MRPS14	FANCE	ALDH1L2
ACADL	SLC6A12	ZNF680	SCO2	ABCB8	MYO1D	NNT
MRPL4	MACO1	ZNF320	NARS2	ATP5F1C	MBOAT1	GLS2
UQCC1	MPZL2	MUC16	ZADH2	PPTC7	CNGA1	GPT2

MRPL55	FMN1	SLCO1B3	TRIAP1	COX5B	ALOX5	FAM162A
MRM1	PLEKHO2	ZNF260	PTCD3	ALAS1	ST3GAL1	FASTKD1
MRPL44	LINC01967	ESYT2	SLC25A16	MTO1	SEMA3F	HADHB
HSDL2	ARHGEF3	BSPRY	SELENOO	NDUFA5	CAVIN2	STOM
ATPAF2	SNX30	PBX2	HSD17B4	NDUFB6	GPRC5B	DGLUCY
NDUFA9	ADA	KIFC1	OXLD1	MTX2	SIX2	PCK2
COA6	EHBP1L1	CMTR1	NDUFA11	FDX1	IGHE	ACACA
POLRMT	XCL1	RXRB	AURKAIP1	TIMM50	EMP1	ABAT
ISCU	DLK2	NPR2	GLUD1	VDAC2	LINC00491	ALDH5A1
RTN4IP1	CLDN1	ZNF875	MRPS33	CMC1	IDO1	PISD
OGDHL	SLC4A11	KLHDC3	MPV17L	NDUFB10	HK1	GARS1
BCKDK	TMEM79	CLDN11	TFAM	ACADSB	TPM4	LYPLAL1
GFM2	HCAR3	CGREF1	OXA1L	COQ4	PKP2	FPGS
NDUFAF4	KCTD3	SLC47A2	PDPR	MRPL24	KRT6B	MAVS
SLC25A35	TPRG1	ZSWIM5	NLN	HSPD1	TRIM6	ACACB
TIMM8B	HR	NELFE	HDHD5	NDUFS5	LMO4	CCDC51
MCAT	LHX6	SERPINB9P1	MMAB	CYCS	ANO1	NSUN4
IBA57	CEACAM6	AARS1	PXMP2	LYRM7	SRPX2	METAP1D
PHB2	SRGAP3	EZR	TCAIM	PCCA	ETNK2	KARS1
DAP3	MFSD4A	CLIC3	MTFR1L	CLPB	CTSD	SFXN2
CMC2	COL13A1	MYO1B	PRELID3A	MRPL36	CCDC162P	FASN
SDHAF1	F2RL1	VTI1B	TOMM70	FARS2	TM4SF1	TXNRD1
MRPS17	PAX9	ATF6B	SFXN5	FDXR	CAMP	BCL2L13
ADHFE1	APOL6	RIPK1	FIS1	GATC	PLAC8	TWNK
NFS1	HIVEP2	ARHGEF35	SLC25A40	TRAP1	CAPN2	FTH1
NDUFAF5	GDF15	IRX3	SLC25A23	IVD	COL28A1	PDP2
MRPL17	NUDT18	SYTL5	SLC25A36	SLC25A5	DEPP1	SLC25A37
MFN1	MARCHF3	AFAP1-AS1	NMNAT3	ETFB	CDH8	SLC25A43
MRPL11	THBD	HTRA3	NADK2	ATP5PB	LIPH	NUDT5
COX4I1	LRG1	CPPED1	FAM136A	COX7C	TIMP2	NDUFAF3

NDUFS6	FGD2	ZNF607	GUK1	CHCHD3	CDYL	OCIAD2
MRPL13	CTSS	C6orf62	ECSIT	ACSF3	BNC1	HINT1
OXCT1	EPB41L1	APOL1	GRSF1	SLC25A10	HTRA1	NUDT9
PDK2	ARRDC4	EYA1	DNAJC30	PDK3	LINC02348	BAK1
LIAS	SPARCL1	RAB15	CAT	CHCHD10	BMERB1	MAOB
MRPS28	PPM1H	WRNIP1	STARD7	CBR4	WDR72	FKBP8
ECH1	GBP4	ANOS1	HTATIP2	HAGH	ABHD17C	FBXL4
BCS1L	P2RY2	TP63	NME4	MRPS16	ALDH1A3	MAOA
ERAL1	SH3BP4	H1-0	GOT2	GPD2	KRT16	BCO2
TSFM	AMDHD1	CYP2R1	OXR1	MT-CO2	FOS	COA7
FXN	GLUL	IL1B	AIFM3	DHTKD1	TMEM40	DHRS2
YARS2	PAQR8	PGM3	MGST1	FAHD2A	AKR1C1	C15orf48
MRPL21	TNFSF15	LCP1	BAX	HSD17B8	GCNT1	PARK7
ALDH4A1	CMIP	SNCA	MTPAP	SPRYD4	CACNA1I	NME3
TOMM40L	GPCPD1	MKRN1	AHCYL1	MRPS26	ZNF488	AGPAT4
NDUFAF1	TMEM52B	ZNF282	ACSL1	BPHL	SYT8	TDRKH
MRPS9	SIRPA	ZNF558	PTPMT1	TOMM40	PXDN	THEM5
COQ8A	NEDD9	TNFRSF10B	PINK1	ACAD10	ALG1L	MGME1
MRPL15	MMP13	INSR	THNSL1	DNAJC11	MN1	AGK
AK3	IGFN1	MARVELD3	ARF5	GLDC	BCAS1	MIEF2
MIPEP	ARPC2	KCNS3	MCUB	SSBP1	CD47	THG1L
CRAT	MPPED2	DOK4	LYRM1	MRPL23	MYB	BOK
PCCB	ST6GALNAC2	DSP	PET100	SQOR	NIPAL1	BCL2L11
MRPS7	OTUD1	PPP1R11	UNG	GCAT	MBP	
MRPL3	FBXO34	BRPF3	TSPO	ECI2	CAVIN1	
PRODH	SPINK5	CASTOR2	UCP2	ATP5MG	SPOCD1	
MCCC1	TPST1	CLSTN1	FASTKD2	MMUT	FOXC1	
MRPS12	DOP1B	FHL1	ABCD1	ATP5MC3	MXRA5	
PDK1	GATA6	RING1	DBI	MLYCD	PEX3	
MRPL49	LIF	FBP1	TK2	NIT2	DPY19L1	

COX7A2	PPP2R5A	CUL4A	ARL2	ME2	LRRC61	
TMEM126A	MYO18A	NOL7	VPS13D	TUFM	GSDMB	
ECHDC2	GPN1	SLC39A7	CYB5R3	TIMM23	BLNK	
HCCS	MARCHF1	TSPAN15	PNKD	RMND1	PDE7A	
HIBADH	PPP4R1	ARHGAP24	BNIP3	MFN2	COLCA1	
MRPL19	GALNT7	CUL1	OCIAD1	NDUFAB1	FOSL2	
SLC25A30	ALS2CL	HSPA2	METTL8	PDSS2	RTKN2	
BDH1	RBMS2	LTBP3	SMIM8	MRPS25	WNT9A	
MTX1	SEPTIN8	ZNF44	TAZ	ACOT13	SDC4	
NDUFA7	PYY2	ATXN1	ACOT11	CPT1A	LYPD1	
TIMM17A	MMD	GPHN	NTHL1	RPUSD3	CALML5	
MRPS18C	ERBIN	SMO	PRDX2	SPR	FKBP14	
ACAD8	STX6	ZFP14	GPAM	MPV17	FAM3C	
TIMM22	ADGRF1	CD38	PRXL2A	MRPL18	STRA6	
MRPS21	WSB1	ZNF204P	POLQ	CHCHD1	CRABP2	
TOMM22	ELF3	NRIP3	LIG3	MPC1	CYP4F3	
MRPL30	TRAF4	ZNF701	BNIP3L	IDE	PTPRS	
MRPS34	ACBD3	LINC01133	CYP24A1	GSTK1	SERPINE1	
ME3	DNAH5	PLEKHG4	PMAIP1	HSPE1	OSMR	
MRPL22	CYTIP	ZNF28	SLC8B1	MRPL54	SLC9A1	
GCDH	FAM198B-AS1	ABCC1	MYO19	MTCH1	PEG10	
MRPL47	LINC02474	PON3	SEPTIN4	AK2	UGT1A10	
PPA2	WSB2	PPFIA3	SPTLC2	NOA1	DAB2IP	
WARS2	LASTR	DOC2A	NDUFB1	MRPL35	AKIRIN1	
SMDT1	RHOD	PNPLA2	BAD	NDUFA3	STING1	
COX7A1	MECOM	RFX5	BCL2L2	SFXN1	SLC43A3	
MTG1	EML4	TBL1X	DMPK	XPNPEP3	KRT14	
COX14	WNT5B	EHD2	STYXL1	SLC25A21	P3H2	
NDUFA12	DENND1B	PRRC2A	SPHK2	SLC25A29	CCND1	
MTERF2	NR4A2	ZNF614	STX17	SDHAF3	UCA1	

ETFRF1	PLA2G10	SNCG	ARMCX6	PRELID3B	SLFN11	
NDUFC2	HIF1A	SSR1		NDUFB3	APMAP	
SLIRP	PRKG2	BST2		C8orf82	ANKRD9	
MT-ATP6	SH3RF1	SLC22A20P		EARS2	CREG1	
TIMM21	IKZF2	GSTM1		GRHPR	NOMO1	
NDUFB11	EXOC6	PRSS12		ECHDC1	DAPK1	
HINT2	ABTB2	SORBS2		HARS2	OCLN	
MRPS5	SREK1IP1	FNDC10		NDUFA4	PSAPL1	
C6orf136	ABCG2	SLC7A8		OAT	CALML3-AS1	
LIPT2	FAM160B2	NUDT3		COX7B	IFITM10	
DECR1	FAM167A	DYRK3		RARS2	ELOVL6	
SLC25A15	AREG	AKT3		CHCHD7	CYP2B7P	
NDUFV3	IQUB	PPP2R2C		SHMT2	CAMK2A	
STOML2	STS	FERMT1		CPS1	ABLIM1	
SLC25A25	ATOH8	TPRXL		MTRES1	TINAGL1	
CCDC90B	TP53I11	SYTL3		TRMT2B	IL1RAP	
ALKBH7	PLAU	TAF11		ACSF2	PPT2	
MPC1L	CNN3	BRD2		HMGCL	KIAA0040	
MRPL27	MCFD2	MATN3		KYAT3	ITPRIP	
HSCB	ATL2	TDRP		TBRG4	FAM83F	
ATAD3A	STARD13	LIMCH1		SLC25A44	LINC02541	
MRPL37	SLC35D1	TRPC1		GSTZ1	TBC1D8	
PTGES2	POR	SLC36A4		PDF	BAG3	
ACSM5	MEIS1	PHF10		IMMP2L	SLC2A12	
CISD1	RNF2	WDR46		MTRF1L	VDR	
MTARC2	HRAT92	NRG1		ACOT2	DNAJC3	
SCO1	KEL	KLC4		SIRT3	BDNF	
UQCRB	GDI1	LRP12		C12orf65	CDCA7L	
ATP5PD	OPHN1	TTLL7		CPOX	ATP1B3	
CCDC58	BHLHE41	GAMT		NGRN	ALCAM	

MCEE	SLC31A2	GADD45B		SIRT5	TGM1	
MOCS1	TJP3	SGPL1		MACROD1	SH3BGRL2	
SLC25A51	TGFB1	SENP6		NIT1	ACAP3	
SPG7	KIF13B	SEPHS1		MCU	NEO1	
OXNAD1	SLC16A3	DDX39B		CRLS1	HOXA3	
DARS2	TMEM123	ASIC1		GLYCTK	TPM2	
PC	REEP4	ACTL8		AARS2	ANXA11	
CLYBL	MLF2	DAXX		FLAD1	FAM20C	
PNPLA8	LGR4	PHF1		MPST	RABGAP1L	
PYURF	PPIAP45	SNRPC		NUDT13	CARD19	
MRPL32	CCNJL	PLIN2		CKMT1A	CYP2S1	
SLC25A26	SNCAIP	EZH2		TOMM5	CRYBG2	
PRODH2	RASSF6	LAMA4		NDUFA1	DOCK8	
FDX2	ZHX2	POLR2C		D2HGDH	LUM	
COX6A2	TNNI2	AKR1E2		OPA3	PPP1R12B	
SLC30A9	SVEP1	ITPR1		MRS2	PAK6	
TAMM41	MICALL1	LINC01278		HINT3	NRP1	
DNLZ	DPP6	NELFCD		DGUOK	TMEM91	
CHCHD4	MPHOSPH6	ZNF777		ACOT9	SCPEP1	
ACSM1	MAP7D2	G3BP1		ACSS1	LINC00857	
COA5	FBRSL1	ADGRL1		CHPT1	UACA	
COQ2	PFDN4	RANBP9		MT-CO3	SH3PXD2A	
EHHADH	BPIFB1	HEBP2		MTRF1	RUNX2	
NDUFS3	ITGA2	ARHGAP40		TMEM143	NLRP10	
ABCB10	SLC41A3	DGKA		TIMM29	B4GALT1	
CKMT2	POU2AF1	COL4A6		PNPO	OVCH2	
ACAD9	AMZ1	RHOB		MICU2	TSLP	
COX16	CYB5R4	RCN3		PCBD2	CA9	
DHRS4	VPS37B	BLOC1S5		QRSL1	VASP	
PRELID2	ARFGEF3	LINC00470		PRDX6	ARFGAP3	

MICOS13	CYP4B1	CCHCR1		NAXD	AATBC	
GLRX2	ZNF697	CMTM6		MT-ND3	FOXK1	
MRPS30	MAPK8IP3	SMOC2		ACCS	EPHB3	
HSD17B10	ARID3B	SLC35G1		PTRH2	FGF1	
SUOX	ADAM19	HLA-C		DNAJC28	OSR2	
SARDH	CTTN	CHST11		METTL17	EPHA2	
RIDA	CYTH4	ANXA8L1		ARG2	VGLL1	
COX8A	PTP4A2	SSRP1		DNM1L	GATD1	
TIMM8A	TRIM56	ID3		FAM210B	KLHL29	
VDAC3	CEBPB	IGSF9		COX20	LDLRAD1	
SLC25A24	COPE	HPS1		HSDL1	FGFR2	
SLC25A22	ANKRD13D	PRKAG2		PGAM5	GM2A	
SLC25A31	SORL1	SMARCA1		TRMT10C	TXK	
SLC25A18	CHPF	BET1L		ACOT7	BTN3A1	
TRNT1	HGS	PHF19		TFB1M	MYH16	
VARS2	ABCC10	EXT1		PYCR2	ACSL5	
MRPL38	ARAP3	STON2		GDAP1	TPD52L2	
MRPL58	TRIM31	TMEM64		MTIF3	TM7SF3	
MRM3	LINC01605	ZNF850		ISOC2	CEBPD	
TMEM70	PYGB	ESRRA		ATP5MC1	PAM	
NDUFC1	ARHGAP23	NPTN		QDPR	CBX6	
PDSS1	MYO1E	GNL1		ADCK2	CST3	
FMC1	MAX	SERTAD2		AGPAT5	LIMA1	
ACSM3	C6orf132	MAP3K7		MCRIP2	VSIR	
NDUFAF6	NAB2	HERPUD1		SLC25A28	RASGRF1	
UCP1	IGSF8	TSPAN7		EPHX2	SYNJ2	
GLRX5	PHKG2	LAMB2		MICU1	FBLIM1	
PTRH1	TCEAL3	RGL1		BOLA3	ANKRD2	
MRPS11	EMBP1	RPRD1A		RSAD1	AKR1C2	
ECI1	CYBRD1	BTN2A1		PEX11B	PIFO	

SLC25A45	MATN2	SPIRE2		TMEM186	MUC20	
SLC25A39	SLC29A1	KCNAB2		TOP1MT	MLKL	
REXO2	PLEC	PIK3R3		RNASEH1	GALNT12	
GATM	ZFP36	TRPM4		LDHB	WWTR1	
TIMM10B	LRFN4	SPSB1		YBEY	COLCA2	
SARS2	B3GNT3	ARHGEF7		MUL1	RPS6KA5	
CHDH	CDC42SE2	WWP2		BID	HCAR2	
MRPL57	OTUB2	ANKS1A		SYNJ2BP	SYT7	
MTFMT	IRF1-AS1	CDC25B		MGST3	CA13	
NDUFA13	PLCL1	CPNE2		ACP6	EDIL3	
TMEM11	FBLN1	TDP2		DDX28	LINC01559	
NAGS	MAP3K5	TBC1D22B		MTX3	SPTBN5	
NDUFAF7	MAST4	B3GALNT1		MUTYH	CDYL2	
MRPL50	ATP10D	SLC16A1		GTPBP6	PLB1	
LACTB2	TMEM171	KRAS		PPM1K	IQCB1	
RMDN1	SERPINB2	ADORA1		MSRB3	SPOCK3	
MRPL14	GRINA	CDKAL1		MARCHF5	FLNA	
RDH13	TPM1	MAPK15		SFXN4	PTPRH	
MRPS24	B4GALT5	SCN8A		TRMT5	GPR78	
COA3	PPP1R1C	TCF25		OGG1	BTN3A2	
ENDOG	BCL6	TCF19		AIFM2	PLEKHA7	
BCAT2	HS6ST2	GMPR2		DMAC2L	CACHD1	
TEFM	MAEA	AKTIP		RMDN3	FADS1	
TRMU	DDX18	ZNF300		TMLHE	RARB	
MRPS22	WDFY2	ZNF585B		TOMM20	ERAP2	
LAP3	SLC26A6	MYADM		MMAA	MBOAT2	
FHIT	CXCR2	DYNC1LI2		MIEF1	PITX1	
NUBPL	IDH1	CSNK2A2		UQCC2	CFAP57	
LYRM2	PRKG1	FIRRE		PDE12	TFPI	
OXSM	AIDA	TRIM26		DHX30	HCFC1R1	

TARS2	EIF5A2	H3-3B		FASTK	SASH1	
ROMO1	SLC5A12	ZBTB22		METTL15	GATA2	
DNAJC15	HEG1	ZNF816		PANK2	CSAG3	
MAIP1	DUSP3	SLC16A2		NT5DC2	SLC46A1	
HIGD1A	ADAM10	DLX1		ATAD3B	LGALS8	
C16orf91	SH3GL3	GLRX		PRKACA	GRK5	
RHOT2	SHTN1	EML1		COMTD1	FYB1	
HAO2	CD274	ASCC1		CPT1C	SULF2	
RDH14	SMTN	GPRIN2		MIGA2	LRRC58	
ATP5MPL	DENND2B	COL18A1		SND1	KRT5	
CA5B	PTTG1IP	RTN4RL2		CDK5RAP1	GDA	
COASY	FOXQ1	ATP1A1		CYP27B1	TIPARP	
CISD3	CYB5R1	HBS1L		BIK	PCBP1	
COX6B2	CYRIA	NEK6		MTERF1	OGFRL1	
GLOD4	TMEM51-AS1	BBOF1		SPATA20	ZNF812P	
ACAA1	AMDHD2	CCDC28A		APEX1	TLR3	
COX19	DLL1	PCID2		ATPSCKMT	H2BC21	
AMT	SLC5A3	FAAH2		DUS2	ATP1B1	
BOLA1	DTX4	ROR2		CKMT1B	EML2	
ALAS2	E2F2	DDX19B		CASP8	NPNT	
GRPEL2	SLC1A3	PCIF1		BCL2L1	EFHD2	
GFER	INTS6L	GNG2		EXD2	RAET1G	
COX4I2	MALL	TPMT		ARMC10	SLC4A7	
MRPL39	STIL	PRPF6		PREPL	MLF1	
AADAT	RBP1	PEX26		CEP89	TIGD2	
FUNDC2	JUN	NUDT16L2P		FKBP10	DPP4	
LYPLA1	NAPA	LARGE2		DNA2	PRR5L	
PDHA2	IER5L	ZNF322		ALKBH1	TRIM25	
GTPBP3	RGS9	SCARB1		NSUN2	SOWAHA	
MTHFD2L	ANKS6	GPR143		NSUN3	CHAC1	

COA8	STEAP3	MBTPS1		SERAC1	HOXA-AS2	
PPOX	ZNF165	UPK3B		SPIRE1	GZF1	
LDHD	HSPG2	MYO5B		POLB	LNCAROD	
MRPL45	FRYL	CCDC184		ARMCX3	LHFPL2	
IMMP1L	SHLD1	SAR1A			GNAO1	
POLG	GPR153	SPON2			FOXF2	
ATPAF1	MSN	EXOC2			NTN1	
BLOC1S1	IVNS1ABP	PLCH2			ITPRIPL2	
COX18	OBSL1	AKT2			EGR3	
NDUFAF8	BTN3A3	SAV1			FA2H	
ATP23	SNAI2	SLC39A8			DENND6B	
DCAKD	LAPTM5	ARMCX4			NRP2	
ATP5MF	ACTB	KIF13A			IRS1	
SFXN3	FAM50A	ANXA10			SRRM3	
AASS	CTDSP1	CHID1			CLDN16	
DIABLO	CAP1	DNTTIP1			DAGLA	
COX8C	BTN2A2	MPP1			TFAP2C	
AGXT2	MUC1	NT5DC1			RNF144B	
APOO	SHE	TEAD2			NFKBIA	
ACAD11	CAV1	LDB1			ADGRV1	
C5orf63	SCNN1A	CASZ1			DNAH10	
HOGA1	RRN3P1	PCBD1			HAS3	
TTC19	ACTG1	PTEN			TLR6	
GCSH	B3GALT5	VAPB			TMEM37	
TOMM6	TRIM16	MSANTD4			HIP1R	
ABCB6	CD40	GPM6A			GNE	
TST	TRIP10	NFKBIL1			RNF152	
MT-CO1	AMMECR1	ZNF133			CBR1	
SDR39U1	MGAT3	ZNF329			CYP26B1	
DMGDH	LINC02428	DBN1			ITGB4	

SLC25A14	TXNDC16	BRD7			TBC1D10B	
CHCHD5	CYHR1	NEFM			CERS6	
FAHD1	GIMAP8	GJA3			GYS1	
CYP27A1	FNDC3A	GLIPR2			ITPKC	
MT-ND2	STK17A	FRK			CASK	
MT-ND4	PPP1R9B	NATD1			MUC20P1	
MT-ND5	AGTRAP	ZNF184			PLCE1	
DELE1	VWF	ABT1			FYN	
MRPS31	NELL2	TRPV6			ATP2B4	
ALDH1L1	MYL9	PPDPF			BARX2	
PTCD1	HSPA1A	TFDP1			SBNO2	
TRIT1	CTDSPL2	ECM1			ARL2BP	
SLC25A32	PDCD6IP-DT	ZFP30			SVIL	
EXOG	RASSF3	PTGFRN			NR2F1	
CHCHD2	MXI1	PDLIM1			SLC37A2	
KMO	EPHB6	UROS			SDR42E1	
ELAC2	DNAJB2	C1QTNF6			NRARP	
GPX1	MBNL3	PLCG2			IGSF3	
CPT1B	ATP6AP1	RSPRY1			TPBG	
PRELID1	PEF1	RIN1			LMO7	
UCP3	TACSTD2	GSTM3			ARHGEF37	
SLC25A27	ABHD3	LRRN2			ARHGAP18	
HEMK1	ALDH3B2	GTPBP2			TMCC3	
OMA1	ASNS	CSNK2B			WARS1	
LETM2	SDCBP	FMNL1			SLC22A23	
DHRS1	SIRT7	BMP7			ALPG	
METTL5	HTR2C	SHROOM1			LFNG	
MSRA	TCEAL1	AGRN			ULBP1	
NUDT8	FMR1	GPNMB			RAPGEF5	
SLC25A38	C15orf39	PDZD8			B4GALNT1	

MPV17L2	WRN	HTR7			C9orf72	
ACLY	CYSTM1	SGCE			ITGAV	
ABCD2	SNHG26	SHOC2			ARHGDIB	
LIPT1	NMU	ZNF212			PHACTR2	
HMGCS2	ASB1	DTX2			SERPINB6	
NDUFB4	PTPRK	CASD1			PTPRR	
UQCR11	UXS1	BDKRB1			PIP5K1A	
HTRA2	REPS2	PCLO			PDCD6IP	
MT-CYB	PARD6G	FRAS1			LAMB1	
AKR1B10	DZIP3	MGAT5			DVL1	
PHYH	PHLDA3	DAPP1			SEMA3A	
NDUFAF2	SERPINB3	ITGA6			CENPN	
PARL	DDIT4	RASSF8-AS1			DAPK2	
MRPL42	LSP1	TRUB1			TLR1	
DNAJC4	ATAD2	VIM			GDPD2	
PAM16	IQANK1	WSCD2			LRATD2	
ALDH3A2	GRAMD1C	BAHCC1			JPH2	
ACSL6	WDR55	BECN1			SLC41A2	
ABHD10	NOS1AP	DUSP22			ARHGDIA	
PXMP4	RAG1	TSPYL4			KIFC2	
OSGEPL1	LOXL4	HLA-DRB6			CLIP2	
NUDT2	SOCS6	MANCR			PREP	
CYB5B	THBS1	TRIM38			GALNT3	
MRPS18B	THAP9-AS1	EPHA1			ATP6V0D1	
RFK	SEPTIN9	MCM9			SERPINB13	
MT-ATP8	LYZ	PRDM5			CDKL1	
TIMMDC1	PIK3CA	GDI2			ELAVL2	
SPATA19	RETSAT	SGPP2			ANKRD13A	
AKR7A2	ADSS2	SLC9A9			VSNL1	
DUT	CFAP20DC	RAPGEF4			EMB	

DHRS7B	GLCE	ITGAX			SH3TC2	
NT5M	PARP14	TMX4			ARL14	
RHOT1	APH1B	LANCL3			MMP14	
MTHFS	MICALL2	WIPF1			MIER3	
ADCK1	PAPSS2	LINC00958			PIM1	
NIPSNAP1	HPGD	IL12RB2			ACER3	
ACSM2A	LINC02028	ATP6V0E2			EDAR	
SLC25A33	ZBTB7C	GINM1			ITCH	
LYRM9	TMEM204	DNAH2			ATP9A	
RAB24	KIF11	TNKS1BP1			CLGN	
PARS2	IARS1	LINC02518			INHBB	
MRPL52	ENDOD1	NAE1			KDSR	
PUS1	TACC1	EHD3			PTK2B	
PGS1	LTBR	GTF2IP4			RBM18	
TMEM205	TRIM34	CERS4			JAG1	
SLC25A53	CORO1B	ANXA7			RBCK1	
MTFP1	LSM7	GALNT11			TMC6	
COA4	FZD5	SYBU			VILL	
NIF3L1	CLOCK	PPIL4			PGM2L1	
PUSL1	MINDY2	CERNA2			LAD1	
SDSL	HLA-E	AFG3L1P			PRUNE2	
SUGCT	CDK2AP2	APOL2			ARL8B	
NRDC	ADO	MCTP1			BMP1	
TMEM126B	CDC14B	ANO4			TRAF7	
ACSS3	H3C6	TTC26			MIR22HG	
RBFA	ALPK1	ZNF527			HAS2	
NIPSNAP3A	GSTP1	ZFAND3			KITLG	
MTARC1	EML2-AS1	EIF4A3			FBXO32	
FUNDC1	SAMD9	CDS2			ANKH	
CBR3	MBNL1	WASHC2A			ZFYVE1	

MTERF4	PDS5B	C6orf47			IL1RN	
NUDT19	PHLPP1	DPY19L2P2			IGSF1	
HEBP1	TRABD	SLC6A11			RHBDL2	
DTYMK	MTR	RNASET2			DTNB	
STAR	LINC00920	PPA1			IGFBP6	
DNAJC19	TUFT1	COL4A5			RASSF5	
OTC	PPP1CA	TPD52L1			LGI3	
COX7A2L	PPP2R2D	NUP93			HERC5	
MTERF3	CKAP2L	RNF146			MOSMO	
TMEM177	FAM111B	TUBB			TMEM125	
FDPS	C2CD5	FOXP4			SYT12	
IDI1	CRISPLD2	LIMS2			NAPB	
TOP3A	CDKN1A	NRN1			LINC01226	
FTMT	FSCN1	CPA4			ADAMTS16	
LDHAL6B	FAM83H	DCHS2			ERLIN1	
MTFR1	NINJ1	RNF144A			SDC1	
MMADHC	IGFL2-AS1	SMAP1			PKP3	
TSTD1	PPFIBP1	CYP4F12			PHLDA2	
OSBPL1A	HELB	CDK19			PTPRZ1	
RECQL4	MPP2	RCSD1			ITGBL1	
GLYAT	TP53	NHLH2			MTSS1	
COQ8B	ERCC6L	DLC1			SPATS2L	
GTPBP10	ARHGAP1	NPM3			PSCA	
TRMT1	RNF139	PRSS16			SH2D4A	
TSTD3	PDE5A	SGPP1			TYMP	
MRPS36	ZCCHC17	ANXA9			APOD	
SLC25A48	SLC29A3	EDNRA			TSPAN6	
SLC25A47	DENND3	CDR2L			KLHDC8B	
SLC25A41	POLA1	DXO			RPS6KA4	
SLC25A34	IFNAR1	MFAP2			GART	

SOD1	RASA1	SATB1-AS1			PTPRU	
SERHL2	DHRS7	LAMP1			CD151	
CHCHD6	TFDP2	NEK10			EPB41L4A	
PIF1	SMPDL3B	ACD			DPYD	
FABP1	ST3GAL5	ARF6			NEBL	
PLPBP	RAP1GAP2	NQO2			PPARGC1B	
PNPT1	CNTRL	ZNF91			PRKACB	
ABCA9	ESCO2	HCG11			RAB11FIP1	
SCP2	C4orf3	ERG28			FZD3	
NT5DC3	GNAQ	HLA-K			KRT6A	
CMC4	IKBKG	SLC6A10P			UBL4A	
AGMAT	TNFRSF1A	STARD9			ADORA2B	
CMPK2	ZFP36L1	CD99L2			COL7A1	
MFF	RHPN2	MAPK11			PRRG4	
DCXR	NUP160	IL6R			NRG2	
AMACR	PKN2	COG4			JPH1	
NUDT6	LINC02614	ACTN1			ACTR3C	
DMAC2	TMEM229B	OASL			AUNIP	
MRM2	ELF3-AS1	CSTA			CXCL11	
DHODH	ITGB2-AS1	PDGFC			PLCD4	
MRPL48	ZNF341	DENND11			SAMD9L	
AGXT	PIGR	HDAC2			HLA-DMA	
MICU3	G2E3-AS1	FAM8A1			TCEA2	
CYP11A1	FRMD4B	ZNF577			CSNK1E	
SMIM20	EGR2	SUSD5			APP	
RPUSD4	MICAL1	CTNNB1			LPGAT1	
NME6	UBE2J2	TMEM170B			CAST	
ACSM4	CCDC92	OARD1			KDM1B	
NLRX1	AAK1	ERBB3			SCIN	
FOXRED1	CD2BP2	CITED4			FUT8	

TFB2M	TPRG1L	KCNG1			FLII	
ATP5MC2	RNF19B	CLDND1			CDH23	
MT-ND1	PRIM1	PSMG4			DCBLD2	
NIPSNAP3B	RHBDF2	FSTL4			TJP1	
SLC25A6	KCNC4	SAYSD1			EDARADD	
SLC25A52	LCN2	DNM1			EHD4	
ACOD1	TLR5	ZSCAN20			GMDS	
QTRT1	MUC20-OT1	E2F3			CYP2B6	
ATP5MD	NR6A1	FBXO41			PCDH1	
PET117	RRAGA	INPP5F			IL20RA	
C15orf61	RNF7	BCL11B			C18orf25	
IFI27	LAMP3	ERG			PLCB1	
CRYZ	SLC10A3	ARGLU1			WDR48	
PYCR1	PLS1	GNAS			B3GNT5	
YRDC	BIRC3	LIMD1			S100A6	
FASTKD5	RUNX1	USF2			ANTXR1	
COA1	DLG1	TSPAN12			S100P	
COX7B2	ERFE	RNF8			PLA2G4A	
MT-ND4L	MPG	CTSA			CEMIP	
SIRT4	OSR1	ZNF273			GOT1	
USP30	PRPS1	EDC4			ADGRG6	
NOCT	GEN1	DOCK11			ANKRD10	
CA5A	ACER2	SEZ6L2			SLC48A1	
TRUB2	RUSC1	CANX			USP14	
C3orf33	DTX3L	RAB40B			ADAMTSL4	
GLUD2	KL	SIMC1			PPAT	
HPDL	EPPK1	PRICKLE3			SHC1	
ANGEL2	ZNF620	EMC10			IL4R	
ANTKMT	ADIRF	SNHG32			RAI14	
UQCC3	LPCAT4	ZNF649			TTC7A	

PRDX4	BIN3	ITSN1			AMFR	
PAICS	PYGL	BRINP3			NT5C2	
MGARP	FEM1B	HLA-DQB2			CNR1	
HDHD3	SKA3	ZNF613			AHR	
CYP11B2	BEX3	GBA2			KPNA7	
RCC1L	IQSEC3	PRRG1			H2AC6	
FAM185A	RGS16	MAP1A			SKA2	
POLG2	RHOV	SUV39H2			NDC80	
MTFR2	FOXJ2	SETD4			THSD1	
GPX4	SERPINB7	ZC3HAV1			WFDC3	
TOMM20L	RABEP2	FOXO4			CASTOR3	
COMT	BRCA2	RSPH3			CD46	
MYG1	STON1	CMYA5			MTHFD1	
OXCT2	KIF21B	HBP1			OSCP1	
CCDC127	RPS6KA3	CRYBG1			LINC00511	
CRY1	CDCA2	DIP2C			SLC18B1	
ACSM2B	NSDHL	RNF223			CD63	
EFHD1	PGLYRP4	TMEM242			SYNPO	
RAB5IF	HOXA1	POLR1H			LIFR	
DMAC1	NDC1	GID8			NGFR	
NEU4	H2BC12	MT-TT			FUCA2	
CASP9	OTUD4	TRHDE			NECTIN1	
MCL1	PAQR7	JDP2			HPS5	
PLD6	GNS	KIF21A			LINC02057	
TOMM34	SDF2L1	AGAP9			HMGN1	
PRORP	NPTXR	RASIP1			TAF4B	
PLSCR3	BLM	KLF8			FRMPD3	
ATP5MF-PTCD1	ADGRF4	SELENOH			TNFRSF9	
GOLPH3	CALR	PPM1A			GALNT2	
FASTKD3	ZBTB44	SYT16			TGFA	

GPAT2	GEMIN5	EMP3			CMTM4	
NBR1	GNA13	LUC7L2			TMCO4	
SETD9	FAM120AOS	FZD1			ALDH1A1	
CYP11B1	TCEAL4	ZNF391			AGPAT1	
CSKMT	VPS28	TMEM30B			INAFM2	
MIGA1	CCDC120	GALC			IVL	
RPIA	KCNK7	PCNA			SGMS1	
BBC3	HRAS	UFM1			H1-2	
BCL2	ANTXR2	SPEF1			DDX5	
BCL2A1	MTF2	ZNF573			KIRREL1	
CASP3	NCAPH	MAF1			IFI16	
MT-ND6	C6orf141	PLPP2			SEMA6D	
PRKN	GPR161	FABP5			KCNH4	
PICK1	FNTA	SBF2-AS1			CEMIP2	
BCL2L10	MAP3K1	ARID4A			PODXL	
AKAP10	CNTNAP3	C4orf19			TBCB	
ADCY10	GPD1L	PDZK1IP1			RBBP8	
PABPC5	NUP205	MBTPS2			GLMP	
PRSS35	PSMD2	CENPT			FUT2	
PRIMPOL	SP1	FZD8			OTULIN	
TRMT61B	PRR5	CPEB4			NUB1	
PDE2A	CARD11	ARHGEF10L			FHDC1	
PLGRKT	ARPC1A	ANXA3			CYP26A1	
ETFBKMT	EMP2	CUL7			PLLP	
RTL10	RAC3	LINC01322			CUTA	
C2orf69	SMC2	KLF13			BRD3OS	
ARMCX2	BPGM	CCNB2			PLEKHA1	
ARMCX1	ARSA	CRNDE			AGAP3	
SNAP29	LEPROT	GPC1			PRECSIT	
METTL4	TIMP1	KNSTRN			NDRG4	

SPHKAP	EPRS1	IFFO2			PSMC4	
NAT8L	SLC22A5	PTK6			GPX8	
MCCD1	MAP1LC3B	SDHAP3			IFIT2	
PIGBOS1	FBXO2	PALM2AKAP2			METTL7A	
HTD2	PPP4C	SLC22A15			ARHGAP42	
RP11_469A15.2	NCEH1	ANXA6			ANXA4	
	CSNK1G2	MORC3			PRSS3	
	STMP1	KIT			EFNB1	
	RTTN	VPS9D1			CCDC33	
	PRKD3	TAF8			FN1	
	LAMA5	FAM149A			TFE3	
	TMEM250	GNG11			SOWAHB	
	MLXIP	CCNY			IL6ST	
	ANKRD28	IFITM2			LPCAT2	
	C2orf27A	CACNG4			HMOX1	
	TARDBP	IL11RA			REPIN1	
	LMNA	CDCA8			CYSRT1	
	MYL6	RBBP9			SLC27A5	
	PEBP1	IRX1			ZSCAN31	
	BACH1	FAM30A			CACUL1	
	ZBED3	TMCO3			SPIN4	
	PLIN3	ZNF266			PYROXD2	
	MITF	ADAM17			ITPK1	
	DNMBP	CEP170B			GNB1	
	RASAL2	NTNG1			CD82	
	DCLRE1B	TCAF1			ADAMTS20	
	IRAK1	KHNYN			APOL3	
	LRRFIP1	ZG16B			MID1	
	BCAT1	CAMSAP3			TMEM154	
	SMCHD1	GLI3			SPATA2L	

	MCTP2	PARVB			RHBDF1	
	OS9	MGMT			PKP4	
	PSD3	MYH10			MED15	
	TLCD1	CCNC			RAB7A	
	PLEKHA2	TMEM38B			ITGA3	
	GNB5	PRKCQ-AS1			ATP13A3	
	CTDP1	PPIL6			RRAS	
	MTREX	ZNF431			ESR1	
	WDR36	PODXL2			KCNK1	
	RTL8C	DCAF11			INSIG2	
	AMER1	NFE2L2			WNT2B	
	SDAD1	TOR4A			NHS	
	CXCL16	SEC63			CADM1	
	MOB3A	CTCF			ANK3	
	MYL12B	CEP57			HECA	
	POLD2P1	CHN1			TMED10	
	SELENOM	RBL2			IRX2	
	ROM1	TWSG1			CDH1	
	PIEZO2	LDLRAD3			CORO2A	
	CNNM3	PGPEP1			B3GNT9	
	RHCE	CEP112			SYDE2	
	GCC2	TWIST1			TMPO	
	ADAM23	STMN1			TUBB4B	
	IPO7	DNAJA2			DDB2	
	PPP1R13B	LTBP2			SRPK1	
	RNF149	C1orf74			UCN2	
	NCOA7	SMIM13			PFKFB4	
	B3GAT3	ADCY3			TGFBI	
	NOD1	TRIM24			GGT6	
	CDK6	GABRQ			KCNJ15	

	NUDT22	GDPD5			OLMALINC	
	FAM102A	GLO1			CAB39	
	KRT3	ASPHD1			TXNDC11	
	NUP62CL	PRPF4B			TMEM132A	
	NUP210	RIOK1			MISP	
	CTSB	FBXO25			CYP1B1	
	HYAL1	ARL15			ABI3BP	
	MED18	TOX4			MYEOV	
	ELK4	BTG1			OAF	
	GNB2	RTL8B			SP3	
	CCDC121	AP3B1			LINC02542	
	TNFAIP8L3	HMGN4			RRP36	
	YBX3	VARS1			C5orf66	
	GPRC5C	XRN2			FMO4	
	CRCP	PBX3			CYB5R2	
	PPP1R18	PKIA			MYO5C	
	ANKMY1	PCED1A			TUBA1A	
	HRCT1	TTC39A			KLK5	
	NCOA3	PEAK1			CDH3	
	ADAMTS1	LIG4			TMEM50A	
	JPT2	PFKFB2			WLS	
	PRKAG3	PCNX4			NAMPT	
	CARD16	LY6D			PPFIBP2	
	LIPE	RHOG			S100A2	
	CCNB1IP1	ADAMTS12			DNAH3	
	STK25	RIPK3			KCTD20	
	SLC4A3	CTAG2			LCA5	
	IER2	HNRNPR			ANKDD1A	
	PALB2	TMEM184B			TMEM156	
	PCBP4	NUTM2A-AS1			FAH	

	TNFSF14	WASHC3			TMEM184A	
	NOTCH2	SLCO3A1			ACTR3	
	ARMH4	POPDC3			PCGF5	
	CSRP1	KRT13			CASP1	
	SEC14L1	LRRC8D			DNER	
	ZNF589	PLXNB1			CYP2T1P	
	TAGLN2	AKIRIN2			PHF23	
	TRIM31-AS1	EREG			FAXDC2	
	SMAGP	PARP2			IKBKE	
	CEP68	ATN1			LRP4	
	HEATR1	ZDHHC5			ERF	
	VRK3	ZNF350			TNC	
	SECTM1	HAUS5			CFHR3	
	PSAT1	DTNBP1			ZCCHC4	
	C19orf12	PTGS2			APAF1	
	SLC12A2	HSPH1			FAM83A	
	APPL1	LINC01224			RNASEL	
	IL17RD	MMP15			ZNRF1	
	KIF3AP1	CDCA7			LIMK2	
	H2AW	STK35			AGR2	
	GNG12	PCYT1B			SUPT5H	
	LNCOC1	SNHG8			CYP27C1	
	ALDH3A1	BEX2			SLC9A2	
	SERTAD1	ADM2			ATP6V0D2	
	LIMS1	XAB2			LPCAT3	
	HMSD	RELL2			THEM6	
	KIAA1671	ZKSCAN8			C14orf132	
	NLE1	PDGFB			SEPHS2	
	B9D1	RNF128			GATA3	
	TRIM29	SMAD7			TTK	

	DNAJB1	FAAP100			MYOF	
	BUB1	RAB26			DLGAP1-AS1	
	CORO6	NBL1			BARX1	
	FOXA1	DHX38			DLG3	
	CEBPA	SLC38A7			LYPD3	
	LY6E	TMEM255A			EPS8	
	POMGNT1	SLC39A6			SYTL4	
	SMPD1	MAP3K4			SERPINH1	
	CAPN8	MARK1			IER3	
	TP53I3	TM9SF3			MCM3	
	EXTL2	FBH1			SLC52A3	
	AP1S1	RASSF9			TP73	
	LINC02029	PARD3			MMP7	
	CA2	SPCS3			MAP7D1	
	CTNNAL1	ZNF33B			AHCYL2	
	SLC12A7	IRF3			CTH	
	MAPK3	LINC00294			ARHGAP26	
	KREMEN1	ST3GAL2			SH3D21	
	UROD	STK19			LINC01589	
	TXLNG	SPINT2			C20orf194	
	ZNF84	HMGN3			DNAJB6	
	PLXNA3	GMNN			UHRF1BP1	
	PKM	MCM3AP			SHROOM3	
	INO80	CERCAM			C1orf226	
	RMND5B	MACROH2A1			MIR4435-2HG	
	CDC42BPA	WDR19			DMTN	
	BRCA1	RADX			PPARD	
	GPR37L1	EEF2K			TRPV4	
	SPTLC3	TUSC1			IL1A	
	ZNF551	DNAJC9			GCLM	

	DTL	BMP2			PLBD1	
	C1orf198	TCP1			SLC35A4	
	IPO5	SLC30A1			FNBP1	
	CARHSP1	ZNF71			KANK2	
	PDE4DIP	WEE2-AS1			RNF141	
	MAML2	CSRNP3			CRISPLD1	
	TNFRSF12A	PRMT2			TRIB1	
	COL6A4P1	ZNF256			PITPNM2	
	ATR	CSNK2A1			ADAMTSL5	
	PRKAR1B	IGFBP2			TCF7L2	
	TAF1A	MMP2			PPT2-EGFL8	
	BIN1	ADGRE2			FHOD3	
	HIC2	TRIQK			INPP1	
	HK2	VTA1			LINC00881	
	PCGF1	ABCF2			PROM2	
	ANAPC1	RHOQ			KRT18	
	TLE5	RASA3			CD9	
	HAS2-AS1	ASB13			CASP10	
	CBFA2T2	RAP1B			SORBS1	
	NFKBIZ	MAP3K3			PFKFB3	
	ADGRA3	TDP1			SIK1B	
	AMBRA1	FILIP1L			MIPOL1	
	SORD	TXNIP			TAPBP	
	LINC02893	LRP10			AMOTL1	
	GDPD3	PLA2G15			GABARAPL1	
	NSD3	ZNF813			KRT16P6	
	NLRP7	PPIL1			CD74	
	RMI2	JMJD1C			SCOC	
	DTX3	ZNF559			GPR39	
	DENND2A	ZNF525			HSPA8	

	JAG2	CRNKL1			RFWD3	
	MDN1	DOCK8-AS1			VPS35	
	FLG	ZNF117			SLC22A18	
	LARS1	CCNL2			MFSD6	
	HNRNPH1	GLE1			DNAJC10	
	DNAJC8	HMCN1			HLTF	
	RIOK3	RECK			ACKR3	
	FAM83B	H19			TSHZ1	
	ZBTB34	UBA2			S100A9	
	MANEA	SLC17A5			PNMA1	
	AP2B1	ATP7B			KIF23	
	FAM111A	ZBTB12			IQSEC2	
	INTS6	PER1			HLA-DMB	
	KIFAP3	ERMAP			ERLIN2	
	BCAS4	NFE2L1			MORF4L2	
	NOL4L	PPP2R5E			SQSTM1	
	MAP2K2	EIF4G3			TGFBR3	
	MLLT6	NOM1			MANF	
	ADAM15	ZNF383			HNRNPK	
	CCDC80	PAX5			ARL4D	
	HLA-F	SNRNP48			TMC1	
	GSAP	TRIM52			CCDC6	
	CAND1	PCDH10			PPM1L	
	CLTA	KLHDC4			VAT1	
	GBP1P1	WIPI2			EPCAM	
	PTPDC1	GCHFR			CDH5	
	KIF4A	LSM2			LBR	
	TOR1AIP2	OR7E14P			XDH	
	HPCAL1	ZKSCAN3			CCDC47	
	SRA1	KCNJ2			CALCRL	

	SERINC5	SOX4			EYA2	
	TGFB1I1	TMEM192			CAPZB	
	ADH1C	APCDD1L-DT			CASP4	
	H2BC5	CTBP2			DEDD2	
	WDR61	CFD			STC2	
	MPRIP	CNTLN			CRELD2	
	ABCC3	FOXO3			TANC2	
	RNF19A	NKX2-8			SGMS2	
	CALHM2	ZNF799			LGALS1	
	RNF185	MAPK12			CTTNBP2NL	
	MIS18BP1	ZBTB2			SSR3	
	IFIT3	UBN2			WNK2	
	GBP7	PAXIP1-DT			MEGF6	
	EPHB2	KIFBP			OGFOD1	
	BTG3	RHEB			TTC27	
	VEGFB	GLB1L2			GCNT4	
	OR13A1	THAP11			PRR15	
	CCP110	CSTF2T			DUSP7	
	CYP3A4	SKIL			APLP2	
	PSMA6	ZSCAN26			PAQR6	
	FBXL14	KCTD12			CFH	
	ARHGAP8	TMEM45B			ZYX	
	RALB	PGAP4			EMD	
	SRSF9	PCMTD2			WDR82	
	PXK	PFDN6			APOBEC3C	
	CLCN7	NAA40			PXN	
	UBE2G1	TYSND1			SH3BGRL3	
	RAP2C	CMAS			ENC1	
	RBM26-AS1	UBE3C			GABRA3	
	FLG-AS1	DYNC2I2			IL13RA1	

	ARL6IP5	DHCR24			ADGRB2	
	MID1IP1	PIDD1			PRNP	
	IPPK	METTL27			SEMA4D	
	RB1CC1	ESPN			WDR12	
	TMEM51	ZC3H18			MAN2A1	
	CEACAM1	GJB7			XRCC2	
	ARFGEF1	ARHGEF5			DCLRE1C	
	WDR1	RAB29			MKNK2	
	PARPBP	DDX27			MILR1	
	SURF4	CNTNAP2			NR2F1-AS1	
	SCARA3	BTBD9			CLCN3P1	
	S100A3	CCNA2			PTCH1	
	HNRNPH2	SLC35B3			CNOT4	
	FNDC3B	VASN			SMAD6	
	FANCD2	PID1			TNFRSF25	
	ROGDI	TMEM254			DNAJC5	
	ANKIB1	SHISA5			EGLN2	
	FIGNL1	GPANK1			ARL5B	
	NRG4	WDR59			RAB3D	
	TMEM41B	CDKL2			GAL3ST4	
	DESI2	THBS2			CORO1C	
	ASAP2	KCNQ1OT1			VNN1	
	SLC9A3R2	PDLIM2			ALDH3B1	
	BCL2L14	F8			MGAT4A	
	NBN	PKN1			SLC2A1	
	CEP70	AOX1			AKR1B1	
	RNF187	GK			PRAME	
	F2R	FOXP1			CLIC1	
	CXXC1	S1PR3			JMJD6	
	SLC26A2	JARID2			FZD10	

	GNL3L	SEL1L3			FTL	
	N4BP3	FIG4			TRIM27	
	NRBP1	PLAT			PERP	
	SLC26A11	KDM4C			CHPF2	
	ANKRD23	EDEM1			XBP1	
	ASPM	AP1G1			MGLL	
	NCAPD2	GAA			SERPINB1	
	HOXB3	TUBGCP3			USP1	
	IK	ID2			ADM	
	SNAPC1	POP1			UBE2Q1	
	SNX17	USP54			HSPA1B	
	OAZ1	WAC			PDPN	
	UGT1A1	GALNT1			KLHL15	
	TAGAP	LGALS3			PTMS	
	PLOD3	GMEB2			MCRIP1	
	FAM118A	BTBD6			DDR1	
	CCDC85B	RANBP6			SESN2	
	MEAK7	SNRPA1			DDX60	
	BRMS1	COL9A3			LARP4	
	HSH2D	TIGD7			PCM1	
	LINC01277	LINC00839			PSMB10	
	LGALSL	PRR12			CYB561	
	PSMC2	DNAJA1			TSPAN13	
	RNF13	LINC00355			TUBE1	
	SLC39A4	VOPP1			HPSE	
	DNAL4	VEGFC			PCSK5	
	MRE11	PARN			DAAM1	
	SLC35B2	PAK1IP1			ZBTB7B	
	UBC	PLCB3			UTP20	
	NTF3	ZBED5			SEMA5A	

	L3MBTL2	PAXIP1-AS2			HHEX	
	TAS2R38	SLCO1B1			ACP3	
	ACBD7	DPYSL2			SIX4	
	PI4KAP2	HSPA4L			SULT2B1	
	PSME2	VAC14			UNC93B1	
	SAA2	ANP32B			PCYOX1L	
	RAVER2	ZNF566			METRNL	
	DPF1	TNFRSF14-AS1			TPP2	
	NEPRO	ARHGEF18			KYNU	
	C3	SERPINB8			ALPP	
	CEP295	WDR44			SOX9	
	TNFAIP1	VPS16			NXPE3	
	RIF1	FOXA2			PSORS1C1	
	SLFN5	RNF166			PARM1	
	JADE1	BCAR3			RSRP1	
	ENTPD8	SRP54			CES2	
	BRI3	HNRNPF			PGGHG	
	DAPK3	CLIC5			RIT1	
	TARS3	NFYA			B3GNT7	
	NUP188	CLDN7			CYP4F11	
	ICE2	NOB1			PANX2	
	FOXJ3	CACTIN			KLHL22	
	LINC02784	LPCAT1			AACS	
	TUBB8	MED22			NANOS1	
	EML6	PTHLH			LINC00885	
	URB1	GNAZ			EXO1	
	MTM1	ZNF354C			LAPTM4B	
	EYA3	IL36G			TFAP2A	
	TPT1-AS1	PNPLA6			MSL1	
	RFC3	CORO1A			COL4A1	

	NRBP2	MESD			MAL	
	LINC00504	PIAS2			DBNL	
	MAB21L3	BMP4			TRAPPC3	
	DCP2	TMEM176B			SH2D5	
	TRA2A	STX5			FZD7	
	HCP5	TRIM47			YAP1	
	RBM3	TCTN3			WASHC2C	
	GNPTG	HCG4			MAP2	
	RPUSD1	RBFOX2			ZNF440	
	DYNLL1	DNAJB12			SIDT1	
	KTN1	TNNT2			MORC4	
	KLK8	TXNDC17			MEGF9	
	PIK3CB	ERC1			PLEKHM2	
	USP28	AR			SSPN	
	TTF2	ZKSCAN4			REPS1	
	GJB3	TRAPPC6B			TRIM35	
	PATL1	DIPK1A			CARMIL1	
	POU2F1	MMS22L			POLR1E	
	LRRC8B	CPNE8			RASD2	
	TMTC3	MASTL			ETV7	
	SMC4	ATMIN			XPOT	
	SAA1	DZIP1			RIC8A	
	UTRN	CCNG1			AMOTL2	
	APBB3	CSPG4			NMD3	
	NCOA5	PPP2R5C			BORCS8	
	FLOT2	DHX37			SF3B3	
	LINC01764	SNRNP70			CLINT1	
	FHAD1	ZFP36L2			RRP12	
	SYTL1	MTHFD2P1			MALT1	
	AP1AR	AMOT			RBM26	

	RAD21	NIPAL3			LMO2	
	CDR2	PITPNB			KCTD11	
	TJAP1	FRRS1			CCDC38	
	RHBDD2	MACROD2			ERGIC3	
	LCAL1	CDC25C			CPNE7	
	PAK1	ASF1A			ATOX1	
	SNX13	RHEX			CHST3	
	RPL36AP39	C10orf95			SPTBN2	
	ZNF428	MICB			SEC23B	
	MYBBP1A	SKAP2			IER5	
	LIN54	HACE1			MED19	
	INO80C	SLC2A4RG			WDHD1	
	WFDC2	ZBED9			ATP6V1D	
	SLC22A4	FOLR3			CLUH	
	JOSD2	FAT2			DEF8	
	AGL	CXorf38			TRIP6	
	KLK7	KRT19			MTPN	
	EML5	ODF2			KCTD17	
	SLC44A1	ZMYND10			CXXC5	
	PSMB7	ANK2			HACD4	
	PIGN	DESI1			GBP5	
	LINC02026	PPP1R26			NCKAP5L	
	PPP1R37	ITGB5			SLC38A1	
	ERAP1	C16orf72			UBR7	
	DCLRE1A	STK26			PRR29	
	WBP11	ZER1			TNS4	
	CEP55	WDR89			NMB	
	VWA2	PGF			ANKRD33B	
	LGALS9C	LY6G5C			SLC39A13	
	EPHA4	TMEM181			TMEM98	

	HS6ST1	MAGEA3			SYT17	
	PSMD8	RBM17			KAZN	
	PYM1	DNM2			ISYNA1	
	TCFL5	CCN1			PHGDH	
	COL12A1	ZNF226			ACTR1A	
	ESF1	BMS1P10			SSBP3	
	PNRC1	P3H1			KLF4	
	GEMIN7	FGFR4			DSC3	
	RHCG	LANCL2			NOLC1	
	DNAJC3-DT	TTC6			DYNLT1	
	DLG5	NCS1			SMPD2	
	BUB1B	MNAT1			NAV1	
	LINC02068	ZNF512B			FAM219A	
	NUTM2D	UTP23			GPS1	
	KIAA2013	ZFYVE21			WNT4	
	AIF1L	SLITRK6			AGO1	
	FGD4	ARFGAP2			FLOT1	
	RHOC	KCNH3			DENND4A	
	NAA80	RAB3B			CCDC134	
	TMEM161A	TUBB2A			BDKRB2	
	ANP32E	C10orf99			UGT8	
	ARHGAP11A	SHCBP1			SIGIRR	
	MAZ	PIM3			PPP6R2	
	LTB4R2	NUDT21			MAL2	
	NCDN	GRB14			SPRED1	
	ANKRD29	KLF10			MFSD2A	
	CYB5RL	CCDC102A			SNTB2	
	PPIB	SH3GLB2			NFASC	
	GTPBP1	FNTB			ARHGEF19	
	MIDEAS	CYB561A3			TNFSF10	

	FAM81A	CYTOR			YWHAQ	
	MED21	SEPTIN6			FAM107B	
	NOL8	CADM2			SMURF2	
	BBS10	ARMT1			C6orf226	
	KDM3B	QKI			RGS14	
	SMC6	HNRNPA1			B3GALT4	
	TFG	TFR2			RALGAPA2	
	RUSF1	STAT6			SLC1A5	
	PELP1	HMGB3			GATA2-AS1	
	MBD1	COL5A2			VTCN1	
	ETV3	MFAP3L			OSBPL3	
	TSC22D2	PTPRA			EIF4EBP2	
	RAPH1	HARS1			NUP43	
	ZDHHC14	ZNF75A			TOLLIP	
	CTNNBIP1	MET			MSH6	
	TCTA	SPOCK1			KRT8	
	POLD3	CCN3			SRPK2	
	EIF2AK3	TMEM255B			UBXN1	
	CCDC192	RPL13A			IL18R1	
	SKP1	RFNG			SEMA3E	
	MTUS1	CDRT1			CIITA	
	SH3RF2	CYLD			TSPYL1	
	COL1A1	CDC40			UBLCP1	
	SNRK-AS1	COG8			OLFML2A	
	PRDM11	GPSM3			FUT11	
	VGLL4	ETS2			FAM53C	
	BMS1P1	USP7			GADD45A	
	PPP1R35	OVOL2			MARCKS	
	UBE2S	DRAM1			MARCO	
	GINS4	PRPSAP1			PAQR4	

	MYRF	CAMK1			POGLUT1	
	VPS37C	GAD1			DSC2	
	SIN3B	ZNF37BP			STX12	
	CNOT1	GPX7			PSTPIP2	
	KIF14	LAMC1			SLC9A7	
	CH25H	SPATA5L1			CTSZ	
	RIN3	SMAD2			WDR3	
	FSTL3	FAT3			GRB7	
	USP37	NOC3L			UTP25	
	SLC44A3	ZFYVE26			SWAP70	
	ZDHHC23	SCART1			MMP28	
	OBI1	ZNF302			SYTL2	
	ARSD	ZDHHC7			RAB17	
	TBC1D31	ARL4A			TENM2	
	ASS1	PLBD1-AS1			PLEKHG5	
	OXSR1	ZNF563			ALPK3	
	MPP7	IST1			ABCC5	
	SOX12	AXIN1			SEMA3C	
	GCOM1	ZNF443			ENO2	
	LRP6	ACVR1			EIF3F	
	PDLIM7	PFKL			BBS2	
	SYNE1	BRI3BP			VPS26A	
	GBE1	WHRN			MAP4K5	
	SLC35F2	IMPA1			PTPRM	
	LRRCC1	SLC28A1			CBLB	
	SASS6	CTU2			PTPN14	
	HOTAIRM1	PYGO2			FAM3A	
	DEPDC1	SNX5			GSTA4	
	FAM241B	EXTL3			ITM2C	
	TMEM120A	GTF3C6			ABCA2	

	CGN	SLC23A1			RYK	
	SKP2	PAF1			DUOXA1	
	MBD3	PBLD			YPEL3	
	DSG2	TRHDE-AS1			KNL1	
	COMMD7	AMPD2			FAR2	
	IFNE	USH1G			EXOC3L4	
	PER2	SPRING1			SLC6A15	
	RRAGC	LYPD6			MAPK8	
	CDC23	CENPBD1			VCP	
	TRMT2A	YOD1			RPS6KC1	
	THAP9	EIF4EBP1			ARHGEF1	
	ZNFX1	PRKAR1A			TSPAN5	
	MRI1	FKBPL			PLD1	
	L3HYPDH	SP6			WDR43	
	SLC30A4	BICDL1			C10orf88B	
	ALKBH5	C1R			YTHDF2	
	ME1	ANKRD13B			RUFY3	
	HUS1	COL6A1			PSMD10P1	
	PPP1R14B	PABIR2			AJUBA	
	FAM120A	MAP4K4			TTLL11-IT1	
	ZNF106	CSRNP2			CD81	
	UFD1	EHF			ZMAT3	
	LINC02086	HNRNPH3			RTF2	
	C12orf45	FXYD5			MOGAT2	
	U2SURP	TANGO6			ABITRAM	
	ZNF286A	STK31			ACVR1C	
	ETS1	HMCES			CLIC4	
	ANXA5	MEX3D			CCDC88B	
	FEZF1-AS1	RBM4			CILK1	
	SLC52A2	DCAF12			FRMD6	

	DUSP9	CMTR2			ABHD6		
	HEXA	MED24			ELP2		
	KCTD7	AMD1			EML3		
	CCDC88A	TAGLN3			MFSD1		
	CLCN1	ZNF767P			SH3RF3		
	ANO6	PARP3			STAT4		
	RFX3	BMP8B			SEPTIN11		
	PEA15	RUNX3			PLEKHS1		
	CCDC107	GIMAP2			GPRIN3		
	GMFB	MAP2K1			MED20		
	PCTP	AGAP7P			PTPN12		
	TNFAIP2	PRKCA			SH3GL1		
	INTS11	SHROOM2			RNF165		
	DYNC2H1	LINC01697			NEAT1		
	SEC61B	WDR27			C16orf54		
	NCAPD3	TCF4			NDST1		
	SNX8	ZMYND11			SOX13		
	ZYG11B	WBP1L			SHKBP1		
	SSU72	MROH6			CDC7		
	SCYL1	WWC3			MLX		
	CLTB	COL27A1			NRAV		
	BANK1	UBE2H			ITGA1		
	FAM111A-DT	RAD51			KIF2C		
	TUBA4A	GUSB			GBP3		
	PLEKHG2	SECISBP2			HSPB8		
	TOP2A	DDRGK1			LPAR3		
	UBE2G2	ESRP2			WDR37		
	CPSF1	PKN3			FMNL2		
	STARD10	YIPF2			KIF2A		
	MIEN1	GNAI1			COL4A4		

	SNRNP40	GABRP			ERBB4	
	AMN	FBXO38			ADRB2	
	ZNF688	ZC3H12C			LHFPL6	
	ABCE1	CYB5A			PWWP2B	
	CLCA3P	ARL5A			NASP	
	METTL1	ZNF137P			HDAC1	
	RAC2	ZC3H12A			BCO1	
	CREB3L2	GTF2B			SELENON	
	CENPJ	TUBAL3			CALD1	
	TEP1	INPP5A			XPO6	
	TXNRD3	TMEM38A			TMC5	
	ADRM1	ZNF367			ACSL3	
	POU3F1	FAM91A1			ATP11C	
	TRIM44	GFOD2			GFPT1	
	TEX101	DHX36			LMAN1	
	TTI1	MEF2D			TALDO1	
	IQCJ-SCHIP1	ZEB1			TRIB3	
	STUB1	CCNYL1			MYLIP	
	MFSD11	IQCM			SLC6A6	
	PRDM1	LAMA2			YWHAG	
	JUP	ASPHD2			UGDH	
	TIMELESS	TBC1D7			TINF2	
	BAG1	DYNLT2			ADAM8	
	EEPD1	SNX3			CDK1	
	DNASE1L1	RSL1D1			UNC5B	
	NKAIN2	SPATA33			ANKRD22	
	CHRNB4	PPP1R14C			CCBE1	
	MSH2	PARG			AKR1A1	
	TOM1	FGFR3			IFNGR1	
	CCR3	ARL8A			GBP6	

	CHEK2	ZNF597			CHMP1A	
	CCNY-AS1	EMID1			BCAN-AS1	
	GAPDH	PLEKHH1			SESTD1	
	MZT2B	NORAD			GALK1	
	KRT31	ANKRD27			MYO6	
	MCM2	CTPS2			PPP2R2B	
	TCEANC2	GRB2			C6orf89	
	EVL	FBXL19			SLC39A2	
	MAT2A	HYOU1			LINC00842	
	NEDD1	GTF2IP20			SSR4	
	SYVN1	TANC1			DKC1	
	DDX21	CNPY3			MLLT11	
	MED14	ZNF430			RPL10	
	ELL	POLR3F			ARPC5	
	GTF2IRD1	CXCL1			NXF1	
	COPZ2	ALX1			MVP	
	DACH1	CDK18			AP5M1	
	KLC1	XYLT2			NOL6	
	AIM2	SERPINB5			GCNT3	
	HOMER3-AS1	CRKL			PDZK1	
	NINL	MORC2			PTPN13	
	TBCK	ZC3H14			RAB11FIP4	
	PDE8A	ZNHIT6			WDR75	
	MME	TMEM127			SLC12A6	
	ADGRG5	ZNF585A			GNB4	
	ASPSCR1	CDAN1			CERS3	
	AHCTF1	LRRC20			FOXM1	
	GMPPA	TACC2			LINC02210	
	FTH1P8	TIAL1			GPR157	
	CHKA	DLX3			MAG	

	COMMD3	C6orf120			TMC7	
	TMEM237	NGLY1			TSSC4	
	SERF2	UNC45A			POLH	
	NAA15	HEXIM1			TRIM5	
	SLAMF7	CEP43			ORC6	
	H2BC8	SMIM27			N4BP2L1	
	JAK2	SLC4A8			AVPI1	
	CFAP300	RNF169			UST	
	HIVEP3	ZNF292			SELENOI	
	LMAN2	RNPS1			SCAND1	
	B9D2	ZNF532			SIPA1L2	
	DICER1	ESRP1			A4GALT	
	SMPD4	FAM149B1			CHMP6	
	RPLP1	PPP6R1			MARS1	
	PLP2	CDK2			EXOSC6	
	HPS6	RPS6KA1			LINC01932	
	TSEN2	PITPNM1			RER1	
	AMTN	SLC39A10			PKIG	
	ATXN10	ABHD13			CSNK1G3	
	SMC1A	INSIG1-DT			PFAS	
	ADAT2	UBOX5			GJB2	
	MKI67	GDF11			NUFIP2	
	MPHOSPH9	HLA-H			TMEM33	
	PFN2	PAG1			NXT1	
	TTC22	HECTD2			PRKAR2B	
	HECTD3	PEPD			ABCD4	
	SMARCC1	IL1R2			MTSS2	
	PICSAR	ADCY1			TMTC2	
	RNF207	LAPTM4A			NUMBL	
	UBE2D3	GUCY1A1			ENTPD5	

	NAT14	CDC42			CAPN12	
	ATAD5	TRIM46			TMEM159	
	TNFAIP8	SLC2A5			HLA-DPB1	
	PPP2CA	AREL1			ING4	
	COL5A1	ZNF655			MALAT1	
	STT3B	ASAP3			SGSH	
	LRCH1	OSGEP			CDK5	
	RAB22A	RPL7L1			STEAP3-AS1	
	EEF1D	ALX4			BMPR1A	
	PLG	DNAJC1			SH3GLB1	
	TMEM45A	ALDH1A2			RNF213	
	CEP290	CACFD1			WASH9P	
	LINC02443	PSME3IP1			ASCL2	
	CDK2AP1	C1orf116			PROS1	
	ASCC3	LCK			NIBAN2	
	EOMES	SOX6			FEZ1	
	RALGDS	CCN2			KCNQ5	
	NSUN6	FGD3			CASP2	
	NUDT16L1	SCAMP5			ZNF775	
	PPP4R4	BPNT2			NEU1	
	PRMT9	LINC01011			ZNF160	
	DPAGT1	GINS3			GRIP1	
	EDEM3	NPDC1			EVA1A	
	APBA1	GALNT6			DGKH	
	KLHL28	HNRNPA3			ATRN	
	PLAAT4	ZNRD1ASP			FER1L4	
	SPOCK2	CLEC2D			MAP3K12	
	SLC66A2	CTHRC1			SH2B3	
	TRIP4	RPGRIP1			PLCG1-AS1	
	CAPNS2	SYT5			SLC8A1	

	DNMT3B	TRIM8			WDR87BP	
	CADM4	AQP10			CD22	
	NANS	DUSP16			KLHL3	
	IRF5	AP1S2			CALB2	
	DUSP1	NKX3-1			ZFYVE27	
	SLC30A7	SLC12A9			MDFIC	
	PLEKHN1	NQO1-DT			RFC1	
	TOPBP1	ATP5MK			ZNF695	
	NCBP2AS2	SUDS3			AOC1	
	PM20D2	TTC31			HLA-DRA	
	EIF2AK2	BRAF			RETREG1	
	SH3YL1	PLEKHH3			PRR13	
	WDR77	MTDH			DKK3	
	ZNF778	PAPSS1			USP10	
	CMTM3	PPP2R3A			AP1M2	
	QPCTL	TMEM147-AS1			TIAF1	
	CLBA1	PPME1			HMG20B	
	TOB2	CLEC16A			SIRPB2	
	NAALADL2	AHDC1			NOTCH3	
	WDSUB1	SLC37A3			MAPRE2	
	PARP4	FAM83E			GLIPR1	
	TG	TRIM32			ORMDL3	
	VBP1	NUP153-AS1			TMEM164	
	SLC2A1-AS1	ADRA1D			CD59	
	SPTSSB	ZNF321P			UCKL1	
	MBOAT7	MXD3			ZNF710-AS1	
	UPRT	SPTBN1			KLHL36	
	RPS6KB2	PRMT5			PRRT1	
	ABCF3	ZNF441			VPS72	
	LY6E-DT	SLC43A2			CAP2	

	NEDD4	FSTL1			ADD3	
	CAMK1D	TSPAN33			LINC00964	
	FAM227B	PRSS21			TMEM160	
	PRMT8	MBNL2			CTNS	
	PPT1	SRD5A1			FKBP7	
	PRPF40A	ZNF570			GLIS2	
	SLC66A3	DBNDD1			PDXK	
	ROPN1L	LSM14A			RND3	
	ORAI3	ALG1			SACS	
	TMEM43	HSF2			ATG16L1	
	TRIM22	MYH14			RABGGTB	
	BRIP1	ERMARD			HACD2	
	NPAT	PLOD2			TUT4	
	SCAI	WTIP			METRN	
	MYZAP	ADGRD1-AS1			ESPL1	
	CBL	MSI2			LMCD1	
	NSF	GTF3C1			CYP2U1-AS1	
	CAPN7	ARMH3			ERCC1	
	OIP5-AS1	GPR85			ADAM28	
	ZNF664	CELSR1			EHD1	
	DOK7	SYNE2			VAV3	
	ARPC3	TULP3			CHST7	
	GTPBP8	WDR62			STAC	
	TRIM14	ISG20L2			SETD7	
	TC2N	TPRN			COPS6	
	SLF2	MCM7			SERPINI1	
	TOR1B	CDIN1			TARS1	
	KLRC2	RCBTB2			MAN2C1	
	CC2D1B	OPN3			FAM177A1	
	VPS33A	PPP3CB-AS1			BFAR	

	LRRK1	GPRC5D-AS1				SUPT16H	
	SEC14L4	LRRFIP2				ZDHHC20	
	AP1S3	DUBR				CTNND1	
	TMEM161B	PPP2R3B				LAMTOR1	
	CACYBP	TM9SF1				ATXN3	
	NUP155	SEC24C				CYP4V2	
	BBX	DLGAP1-AS2				KLF6	
	LINC01547	PNPLA4				RRN3	
	MRTFB	RGS17				NR5A2	
	RAB6A	SH3KBP1				PSMB1	
	EIF2S3	BTBD19				DNAJB11	
	MAGI1	KATNAL1				UBE2E2	
	OGT	CAPN14				ZNF879	
	HOXA-AS3	JRK				BARD1	
	MB21D2	PIGC				KIFC3	
	ATG4A	FANCA				PAPLN	
	PPP1R16A	GPATCH4				TCP11L2	
	VMP1	MED23				HLA-DRB5	
	TANK	SLC7A6OS				INPP5B	
	ATP6V1E2	TRIB2				ROR1	
	STX8	MYC				TEX2	
	MIR23AHG	SELENOW				HS3ST1	
	H4C8	TMEM80				SEC14L2	
	HOOK1	ACP2				ATXN2L	
	ZNF618	PCED1B				MMRN2	
	DMXL1	LUZP1				RGL2	
	POLR2B	SV2A				TMUB1	
	GCA	ASPH				ADAT1	
	POLR2E	CTDSP2				MPP5	
	KIF24	SERINC1				TAPBPL	

	C7orf50	PSMG2			CCDC74B	
	BRK1	WFDC5			TMEM63A	
	RTP4	BTBD10			FAM219B	
	RNF20	GLDN			NUP153	
	LINC02577	NQO1			ZNF217	
	CPTP	GMDS-DT			MLH3	
	APOBEC3F	RBM11			HDAC9	
	RNF157-AS1	REV3L			COL17A1	
	TRMT112	C16orf70			CEP83	
	DIAPH1	CFDP1			GOLM2	
	CLPTM1L	CGB7			TMEM201	
	UBL7	PCDH7			KLK4	
	TSR1	TNS1			ELOB	
	MAP3K14-AS1	NR1H3			SUN1	
	SETDB2	CERK			PEX10	
	NOTCH1	TERF2IP			AFDN	
	BCL7C	JRKL			CDKL5	
	ZNHIT1	MIR205			EXOC8	
	JAK1	DDX1			THRB	
	ADCY4	FBLN5			NAP1L4	
	PRXL2C	PINLYP			RBM15	
	PGLYRP3	DAP			TOR1A	
	SCAF11	DDX19A			CPA6	
	ZNF785	ZNF433			IL15	
	LRRC41	MT2A			ENO3	
	CEP152	CPAMD8			ZNF362	
	NAA10	FAM204A			NPSR1-AS1	
	CLK3	ABCB11			RASL12	
	PLEKHM1	NFATC1			CD99	
	ASAH2B	AKAP12			SLFN13	

	PTPRJ	HLA-DOA			STK17B	
	OTUB1	SYNGR3			UBE2E1	
	E2F8	STAC3			KRT15	
	UBE2L6	ZNF398			NGEF	
	PMS2	ROBO3			SNN	
	EDF1	PRKD1			PSMC1	
	HEATR5A	HMOX2			DYRK1B	
	DAZAP2	CENPB			ANGEL1	
	H2BC19P	DCBLD1			TMEM234	
	TMEM199	DEAF1			SLC7A7	
	LINC00472	SPECC1			RAB1A	
	WDR41	ZNF549			TLL2	
	CENPF	RNF212			RP2	
	AFAP1	GINS2			AP2A1	
	CCNF	AMPD3			PIK3CD-AS2	
	LRBA	PVR			RALGPS1	
	TMEM130	MIRLET7BHG			LYPD6B	
	FAM135A	ABHD4			ORC3	
	SNORA59A	MEGF8			RAI1	
	SDF4	CKB			RTN2	
	PSMC5	KRR1			ITGA4	
	GEMIN8	INPP4B			RASGEF1B	
	MUC15	MINPP1			TAP1	
	TMEM134	MAGEA4-AS1			ADGRE1	
	NUP107	PAX6			MAPK6	
	MAPRE3	PRMT7			MEF2C	
	SOCS3	SUCLG2-AS1			S100A8	
	NUFIP1	DPP7			ENAH	
	SLC39A11	MAMDC2			RNF5	
	SERTAD4	ELOVL7			IL31RA	

	SLC9A8	TAF9B			DIP2B	
	DIS3L	PARD6B			BNIPL	
	PADI4	C12orf66			EVI5L	
	CDSN	PSMF1			SDR16C5	
	RCN1P2	DRD4			FANCC	
	POFUT1	MDH1			BRD8	
	NR2F2	JUND			JCAD	
	ZBTB4	HOXA7			NUDCD1	
	ANKZF1	AURKB			ST13	
	RPS27L	ARID1B			RASA2	
	DYRK2	ATG14			KRT8P3	
	UBR5	PLAAT3			PHTF2	
	RBMX2	BICRAL			CAMK2D	
	NEK2	SPNS2			CNTNAP1	
	SUV39H1	IFIT5			MAP3K2	
	UVRAG	SPRED2			HLA-B	
	HIC1	DIAPH3			C11orf68	
	RPS28	TMCC1			RAB25	
	SAT2	ERICH2			JADE2	
	PLSCR1	MANBAL			RBM42	
	MIR9-3HG	DNAAF3			SPG21	
	MPZL1	KANSL1L			RPP38	
	FANCI	SORBS3			ZSCAN12	
	IP6K2	BORCS7			HOXD8	
	PTOV1	PRUNE1			OPTN	
	NHP2	NOD2			MAP3K21	
	NCOA2	RANGAP1			LINC01588	
	C15orf40	DOCK1			TGM2	
	MYL6B	ATP11A			CCDC25	
	NAGLU	ITM2B			SREBF2	

	SLC4A1AP	EEF1A1			TCERG1	
	ATP6V1E1	SLC2A8			ZNF746	
	UBL3	PML			FBRS	
	MEP1A	ZNF14			HDAC6	
	ARF1	CAMTA1			EFNB2	
	STK4	RUSC2			RASSF2	
	RPS19BP1	EIF2B2			MT1X	
	FAM174C	HDGFL2			NUP98	
	APRT	HCLS1			PCSK6	
	PSMD4	CENPU			ZCCHC7	
	SNX22	STX7			LNX1	
	STEAP2	RPL36A			FGF22	
	MDM1	MARK3			DDO	
	INPP5D	PRMT1			MYORG	
	GOLPH3L	HES4			MAFG	
	NEK9	SNHG12			ZNF615	
	CBX5	PPP2R5D			LRRC8C	
	FLJ42969	USP25			SLX4IP	
	AKNA	LEPR			CPZ	
	MZT2A	B2M			RPLP2	
	PADI2	TFAP2A-AS1			KRT17	
	TRPM7	BCL10			ARHGAP12	
	TMEM47	SMPD4P1			IFT43	
	LRRC8A	RIMKLB			ADARB2	
	CCS	SPATC1L			ERVMER34-1	
	TUBB6	LIMK1			AP2M1	
	METTL9	LMBR1			ZNF224	
	PLPP6	PHKB			MDK	
	GJB5	MOGS			DFFB	
	SIN3A	PLEKHF1			FN3K	

	LINC01978	CALCOCO1			YWHAB	
	AP3D1	PHETA2			EXPH5	
	TRIOBP	KIAA1586			DCAF13	
	SHARPIN	SMARCD1			DDOST	
	ZC3HAV1L	ATG12			TENT5C	
	POU5F1	PLCD1			UFL1	
	CEP192	ICMT			ZNF121	
	IPO8	UGCG			DLGAP5	
	DGCR2	CDC123			SEMA7A	
	NWD1	SAMD15			NOL3	
	HSPA5	IRX5			BMPR1B	
	IFNAR2	ATG101			MBTD1	
	CHFR	SBNO1			NCR3LG1	
	SSH1	ZBTB47			ARSJ	
	DRAP1	HDAC5			RNF41	
	IRS2	LSM14B			KDM4A-AS1	
	CDK17	PIGT			CSTB	
	DNAJC13	ATP8B3			PLPPR2	
	RERE	BTBD11			GDE1	
	SCUBE3	TMEM138			ARPC4	
	FOXN3	CDK10			H2BC11	
	QSER1	CEBPZOS			NECTIN3	
	MXRA5Y	CCDC167			FLJ20021	
	MAPK10	PKD2			TLE3	
	FXR2	BLOC1S3			SIGMAR1	
	RCBTB1	ZNF571			ACBD5	
	ULK1	EIF4G2			ASH2L	
	MCM6	USP13			ZFAND5	
	TMPO-AS1	KIF3C			SEC24D	
	TRMT13	PDCD2			SNRPB	

	ATE1	CBLC			PLAA	
	SEL1L	EPN2			IPO9	
	TICRR	RNF6			BASP1	
	RHOBTB3	AP5S1			MBD6	
	SLC22A18AS	PTENP1			PSMD13	
	FAAP20	LRRC23			MAGI3	
	NFE2L3	FLVCR1			KMT5A	
	SECISBP2L	ZNF512			ATG5	
	PLEKHA8	TBC1D14			KIAA0930	
	CEP57L1	PNLIPRP3			ILRUN	
	ZNF658B	LRRC37A4P			MNS1	
	CEL	CAPN3			KLHL9	
	LINC01465	CAMKMT			MRC2	
	ZBTB33	CNOT3			EIF5	
	DAZAP1	SH3BP5			PCGF3	
	KNTC1	TROAP			HSBP1L1	
	TMPRSS3	STIM1			CARS1	
	MTA2	MOB1A			RGS3	
	SPAG4	CD27-AS1			SP2	
	ZNF311	UHMK1			NHSL2	
	RNU6-140P	PCSK9			POLR3D	
	GVINP1	AGAP2			TSPAN2	
	FAM214A	SLC7A6			GSK3A	
	ITGA5	POC1A			PTDSS2	
	DDIT3	ARL13B			ATP6V0E1	
	COL4A3	NCCRP1			ATP2B1	
	ROPN1B	SCGB1A1			BTBD3	
	BROX	C5AR1			ICOSLG	
	TIPIN	RFFL			RARRES2	
	PSMB9	CALM3			SEC61A1	

	TRNAU1AP	POMT1			AK9	
	LRRC56	PAK4			TBCC	
	ZMAT5	PHYKPL			BZW2	
	BCAP31	MTAP			VPS36	
	KRTAP4-1	CEPT1			NTAN1	
	FBXW5	MTCL1			DENND2C	
	KRT4	BFSP1			CTR9	
	BTBD8	UPF2			S100A14	
	C1orf109	TBP			ZFP90	
	STEAP1	GPR176			MIR31HG	
	LINC00174	TLR2			SNRPB2	
	FAM89B	LSR			RAB1B	
	RPL29	CYBA			GMCL1	
	PIGW	TAB2			CIP2A	
	CKAP5	ARHGEF26			ZNF675	
	ZNF786	TRPS1			ZBTB9	
	EHBP1	RPP40			HRK	
	DCTN3	FGR			ATP13A2	
	TPI1	CXCL2			AP2A2	
	CDC42EP3	CDKN2A			ANAPC16	
	RAB3IL1	USP39			SLC16A7	
	CCDC85C	AIMP1			BBIP1	
	NPLOC4	PROSER3			EEF1E1	
	C5orf34	ZHX1			TNS3	
	NCK1	RIPK4			C19orf71	
	LINC02863	PPP5C			DHX16	
	COPS7A	INPP5J			ZBTB38	
	RAB3GAP1	NIPAL2			CASP8AP2	
	LINC00592	AGFG2			LIPC	
	GTF2H5	P3H4			APOBEC3G	

	SERTAD3	KLHL42			PIGU	
	TADA2A	GHDC			EIF3A	
	MUS81	ULBP3			CHMP5	
	ECT2	GNL3			NSUN7	
	BTC	FAM102B			KANK1	
	TPT1	SARS1			PSMD10P2	
	NFATC2IP	MED6			TAF6L	
	RBM41	CSRP2			LAMB3	
	NENF	TNFAIP3			UBR2	
	TCEA1	C10orf55			ZNF714	
	VAMP8	FOLR1			ORC1	
	UBXN2A	TAF1C			CHASERR	
	CCDC50	CELF1			CC2D2A	
	STEAP1B	NELFB			CORO2B	
	RHOA	ZUP1			FHOD1	
	TLCD3A	IGSF10			TTC13	
	BLOC1S4	ACTR2			ARL6IP1	
	MYPOP	MTMR9LP			SLC6A8	
	MED14OS	SPATA6			IQGAP3	
	RNU6-1161P	TRADD			CDKN2AIPNL	
	SIX5	SACM1L			ELF4	
	HNRNPH1P1	POLE2			SMURF1	
	SPTA1	KIF3A			TESK2	
	COG5	ZNF69			UBE2J1	
	FAM86JP	NPM2			CIDEB	
	LINC00880	FUT4			TENT2	
	ZDHHC13	TRIM4			XPR1	
	CNBP	ATF4			ATP2C1	
	CYP2W1	CNPPD1			ALAD	
	RCE1	HERC2P2			LINC01193	

	SFN	ESS2			TNNT1	
	RPS16	PGBD4			CSE1L	
	KSR1	ELOVL4			DEGS1	
	CALM1	TRIM7			PIK3CD	
	ZNF593	B4GALT4			DRD1	
	TAB3	TBC1D9			RPS19	
	ABHD14A	CEP164			CAMK2G	
	TXNL1	HIF1AN			IL15RA	
	TRIM55	YEATS2			DMKN	
	EEF1A1P12	MAP3K6			ELFN2	
	ARHGEF2	RBM33			C11orf80	
	ALG10B	ANKRD40			MEIS2	
	GNPNAT1	IDS			PREX1	
	MAPK1IP1L	ZNF548			DHFR2	
	COMMD9	ZNF606			ZNF750	
	UBL5	TCEAL8			ZBED5-AS1	
	CRYZL2P-SEC16B	NHLRC1			LMNB1	
	TAF5L	NCBP2			CENPO	
	ATP6V1F	MYO15B			CLUAP1	
	ORMDL2	IQCA1			TAF3	
	HS1BP3	KIAA1217			HMG20A	
	PMEPA1	NPY1R			FRMD8	
	S100A16	KRBA1			FAM131C	
	MYL12A	JMJD1C-AS1			P4HB	
	BEND7	MT-TW			CDK5RAP2	
	SIK2	TMEM214			YIPF6	
	RSRC1	CLIP4			GAN	
	MIB2	MAP10			TMEM203	
	SLC12A8	RNF43			RPL13	

	POLR3H	CYP2J2			CCL5	
	OSBPL9	ANK1			NBDY	
	PIGX	ZCCHC3			CA12	
	NUMA1	FBXL19-AS1			GPSM2	
	SLC38A6	HP1BP3			STK38	
	POLR2L	LINC02328			CPM	
	NFRKB	RPF2			SORCS2	
	YWHAE	NKX6-1			IGFLR1	
	RAP1GDS1	JMJD8			ZNF451	
	PCDHGC3	FZD10-AS1			WTAP	
	GLRX3	RTP3			OSTM1	
	GTF3C4	NFATC3			CPD	
	TTC28	MAP3K8			PPFIA4	
	TNK2	SLC38A5			SUSD4	
	THOC2	AP3S1			GALNT18	
	ABCA13	LPAR6			CDT1	
	SYNJ1	MT-TD			CAPG	
	RNFT1	RBBP7			SNX9	
	HDAC4	UGGT1			PADI1	
	ZNF622	TMPRSS13			ERI1	
	SMARCAD1	RPS5			KATNB1	
	PSMA7	MVB12A			FAR1	
	MKLN1-AS	BMS1P23			C16orf87	
	C4orf47	DIO2			FARP2	
	PARP1	DEPDC7			CFL2	
	KRT89P	TICAM1			RGS19	
	GSE1	EDRF1			SNTA1	
	CAD	EWSR1			ISL1	
	SAP30BP	ADIPOR2			CAMKK1	
	PRDM15	KIN			DUXAP8	

	POLR3B	ZNF574			POLR1A	
	U2AF2	SALL4			MEF2A	
	ZNF146	GNPTAB			NBPF9	
	CNOT6L	DNAI4			FDFT1	
	ATM	ITPR3			CALU	
	C4orf48	SCN1A-AS1			DEF6	
	PDIK1L	PRMT6			UFC1	
	TADA3	RAB11FIP3			SMIM29	
	AFAP1L1	SHB			COBLL1	
	TOB1-AS1	FMNL1-DT			DCN	
	STRIP1	SERBP1			SMU1	
	LPAR5	FAM53B			CYFIP2	
	ACOXL	AP3M1			UBA7	
	KIF20B	FBXL8			BHMT	
	POLR3C	POP4			CD99P1	
	SNAP23	RARG			H3C8	
	PAN2	LANCL1			MAP7	
	PITPNM3	NSMCE1			CUTALP	
	ALYREF	ZNF462			F12	
	ICE1	ERCC6			UGT1A6	
	LINC01686	HCG18			MEST	
	PARP10	PURPL			HSP90B1	
	SRF	MKS1			TRABD2A	
	C2CD3	LRRC37A11P			TUBA1C	
	TET2	SHISAL1			AKAP8L	
	S100A11	ATG9B			ZP3	
	TSC22D4	CD2BP2-DT			TXNL4B	
	IFT20	MMP24OS			DDAH2	
	AKAP6	NIBAN1			STAT5B	
	ETV7-AS1	CDH19			HNRNPL	

	PIN1	LLPH			RLIM	
	CNGB1	TRAPPC10			PLCH1	
	STAMBP	USP18			NCAPG2	
	FABP3	ZNF195			ICAM1	
	RPRD2	MOB2			KLHL24	
	KRCC1	HMGN2P46			E2F1	
	NCL	MDC1			MED12L	
	EIF4E2	CCPG1			CTNNBL1	
	LINC00412	SLITRK3			ZGRF1	
	NOSIP	TERF2			IRAK3	
	CTSH	DCAF5			ZDBF2	
	SFT2D2	ZNF600			CHMP2A	
	ZNF678	LYPD2			CROCCP2	
	ANKRD39	ADCY7			CALCOCO2	
	LBX2	GINS1			ANKEF1	
	SELENOK	SLC40A1			WASH2P	
	MYLK-AS1	SMARCB1			CUL2	
	RPL27A	LDC1P			UBXN6	
	CHD7	GRWD1			RBL1	
	PTAR1	ZNF22			DHX40	
	MCM8	AGBL5			PRKG2-AS1	
	INAVA	CASC2			TRERF1	
	C1orf216	RPS15A			DGAT1	
	UBE2Z	FCF1			MAN2B1	
	QTRT2	DUXAP10			ANKMY2	
	RAD18	GASAL1			GLG1	
	SPPL2B	LAMC2			ZNF385A	
	C1GALT1	SCFD1			FNBP1L	
	TMEM240	LAYN			LGMN	
	COL9A2	RAF1			MAN2A2	

	STXBP2	RABL6			AAMP	
	FGGY	KAT5			CROCC	
	STAG3L5P	NKX2-1			ACTR10	
	PLA2G4D	DCUN1D3			MCC	
	TXN	HLA-V			TM9SF4	
	SPG11	WIPI1			MPPED1	
	PGP	ZNF566-AS1			CCDC130	
	ZNF513	MSH5			BEST4	
	FCHSD1	ZNF706			PAXIP1	
	NR2C2	CENPW			CNNM2	
	HERC1	SPC24			SATB1	
	GLB1	CFAP20			RARA	
	AZIN2	YIPF3			BLOC1S2	
	MYCBP2	ZNF502			TLCD4	
	NEDD4L	MAST2			DSTN	
	L3MBTL2-AS1	LRAT			OTUD3	
	HYAL3	ZNF529			STK38L	
	SLCO6A1	ABCA10			COPS5	
	GSDMD	NIPAL4			MAPK13	
	ASXL2	MIR503HG			AP4S1	
	LSMEM2	QSOX2			MAN1B1	
	TASOR2	SLC36A1			UBE2M	
	PRKDC	MON1B			RNH1	
	TTC39B	PPP1R26-AS1			HLA-DRB1	
	RAB34	LRRC1			HOXA13	
	DNAJC7	TBC1D8B			OTUD5	
	ATP6V1G1	LATS1			ATG13	
	TMBIM1	FTO			NBPF15	
	EXOSC5	TMED1			FAM110A	
	BAZ2B	TCIRG1			EBLN3P	

	SMC5	PRX			PTDSS1	
	RPL36AL	TUBBP5			SORT1	
	SRGAP2	NKIRAS1			EFR3A	
	RPL8	GNG4			PDIA4	
	CCN5	GSK3B			HS2ST1	
	PDIA6	CASQ2			PGD	
	ZNRF3	SLC6A9			AHCY	
	FAM76A	PIGB			SLC39A14	
	GADD45G	MIR9-1HG			DEK	
	SSH3	RELB			DNAAF2	
	KNOP1	PJVK			ARL3	
	SPEN	SLCO1B7			ZFAND6	
	GALT	HOXA2			GGCX	
	RACGAP1	PMS1			CXCL12	
	MAMLD1	WASH3P			AP2S1	
	SLC20A2	C6orf223			LARP4B	
	KRT8P39	CCDC8			SLC39A3	
	TPR	TMEM248			EGR1	
	SMYD2	SLC9A3R1			H2AC11	
	MIR570	NPAS2			FAXC	
	C19orf53	PCAT7			GNG5	
	PCBP2	EPM2A			LPIN2	
	FAM3C2P	FERMT2			GCH1	
	SEM1	FBXO3			PPP1R3G	
	MED12	RTL6			COL4A2	
	MIR590	MAB21L4			VPS41	
	CBR3-AS1	TNFRSF19			GALK2	
	EIF3D	KCNT2			ZBTB5	
	LINC02454	SAMD4B			RPL13P12	
	CIT	ODC1			TMEM230	

	USP2-AS1	ACTL10			FAM110C	
	TM4SF1-AS1	IKBKB-DT			CUX1	
	STK11IP	NUDCD3			VPS51	
	CBLL1	RGS10			MAN1C1	
	GATAD2B	WASH8P			ZNF318	
	BHLHE40	SUSD6			DFFA	
	ZWILCH	RIPK2			MDH1B	
	FAU	SELENOF			SREBF1	
	MAF	DCUN1D4			LINC02354	
	ARSG	DDX24			ANXA8	
	CIB1	JADE3			RPL18	
	MYO1C	ZWINT			CD164	
	ZNF546	DNAH6			MYPN	
	ASAH1	BUD13			RPS11	
	MFSD12	FKBP11			LINC01615	
	KLHL17	MT-TQ			MACIR	
	ERCC2	IL20RB			PPP1R12C	
	VPS18	CFAP43			SPIN1	
	CKAP2	PLCXD1			DEPDC5	
	RUSC1-AS1	GNPDA2			SYNM	
	KIAA0513	NECAP2			SLC44A2	
	HBEGF	CIZ1			GSN	
	EPS15	DNAJB4			GPR68	
	POLE	MAP1LC3A			MIR205HG	
	DDX59	CYP2U1			KRT7	
	LINC02041	SESN1			PAIP2B	
	CCDC171	CHAMP1			INF2	
	COPS9	SCN3A			RBMXL1	
	MED31	UBE2C			HIRIP3	
	ARFGEF2	FAM181B			EPS8L2	

	KLK11	MED29			MAP3K9	
	ATF6	GRN			PPP1CB	
	CERS5	LINC00898			APC	
	PAFAH1B1	CYP4A22-AS1			UBE2F	
	ZSWIM1	UBFD1			PPP1R14B-AS1	
	TMEM67	NPR1			TEAD3	
	ZFYVE16	HAGHL			CD55	
	LIF-AS2	APBB2			ACVR2B	
	SRC	ADAM11			FCGRT	
	MGAT1	PSKH1			FAM104A	
	PEX1	ITFG1			FOXK2	
	AP4M1	HSD17B12			LUC7L	
	SWT1	REXO5			MAPKBP1	
	POMZP3	ILF2			CASP7	
	PYCARD	BCL11A			ENO1	
	EIF1AD	BCLAF1			RNF114	
	FAM86DP	EMC8			ARHGAP17	
	SUMO2	DENND5A			FCSK	
	PDS5A	ATP6V1A			FBXO30	
	CEP78	ABHD16A			CARD14	
	ISY1	NR2F6			EMC1	
	GNGT1	CAMTA2			STX2	
	RDH11	NEXN			ZBTB18	
	FITM2	MAGEH1			PDCD11	
	RPS2P25	PCOLCE2			FIGN	
	CYBC1	SLC35F5			SLC1A4	
	TTL	ZNF358			GOLGA7B	
	CEP126	HCG27			TTYH3	
	ADAR	EEF1AKNMT			PCMT1	
	UCK2	RPSA			MAP3K20	

	UCHL5	JAGN1			CCDC88C	
	RGS20	EFCAB12			CAV2	
	ANAPC15	SYT14			MTMR10	
	TPCN1	SLC2A9			STRIP2	
	DERL2	IPO13			CNKSR1	
	EFCAB1	CCDC144NL-AS1			NXN	
	TTC7B	USP21			FAM98C	
	KPNA4	RFTN2			KRT87P	
	VASH1-AS1	COPS8			ECD	
	DBF4	SNX14			GRAMD4	
	SLC33A1	FBXW9			SIM2	
	ING1	CDHR1			BMF	
	TMEM218	CTBS			ARRDC1	
	METTL14	MAGEC2			NCAPG	
	CBFA2T3	TLN1			SAMD8	
	ITGB2	ZBTB25			SMIM3	
	TGFBR1	TUBA1B			PPP3CB	
	CARF	PRKCH			RBBP6	
	CRYM-AS1	ADNP2			E2F6P4	
	UTP11	GPR132			RAB7B	
	IL6	ACTA2			ATF3	
	DRG1	NEIL1			PSMD7	
	SMAD1	SAPCD2			SIRT2	
	MPI	SF3B5			SLC2A10	
	ZC3H13	ENTPD6			CSTF3-DT	
	SNF8	GSTO2			STARD4	
	TTC30A	FAM171B			MUSK	
	NFKBIE	STOX1			SNRPD2	
	LINC00886	SH2B1			TNFRSF10D	
	TCAF2	CBFB			SETD6	

	LAGE3	VPS4A			POF1B	
	SLC35G2	HS6ST3			CAMK2N1	
	MIR3682	PEX7			CPE	
	DQX1	TUBG2			PXDC1	
	RPTOR	PTAFR			UGGT2	
	MAK16	TRNP1			INPP4A	
	RBAK	UNC5B-AS1			GRIN2D	
	AGAP2-AS1	PIAS4			TDRKH-AS1	
	LINC00973	ARNTL2			RBM38	
	DSCC1	RNF14			SINHCAF	
	ATRAID	RAC1			C5orf38	
	SLC35A5	LAMP2			HJURP	
	NBPF20	TTC5			SYT15	
	CAPN10	ARFGAP1			ELOVL1	
	CEP85L	OGFOD3			OPRL1	
	FAM20B	STARD3			MAGEA4	
	ST14	PSMD5			TTC9	
	MEX3A	RTRAF			THSD4	
	ZNF580	LENG9			ISG20	
	CES4A	ZNF710			CDCP1	
	RBPMS	CBLN3			NCOA1	
	FAT1	RIMS3			SERPINB9	
	GSPT1	CDK9			PLD3	
	LTB4R	CIAPIN1			MFSD10	
	AGTPBP1	ZNF708			ANKRD44	
	ARRB1	FAM21FP			TTC21B	
	ODAD4	TAF1			PMM2	
	GOLGA6L9	TAF15			RAB18	
	DPY19L4	NAPRT			TEF	
	TTC23	DHX32			EIF2S2	

	RBM47	EXOSC1			SLC16A5	
	PLXDC1	SHPRH			WDFY3-AS2	
	TIGAR	HEYL			MGA	
	POU2F3	MTA1			FGD5-AS1	
	PSMB5	SYCP2			Y_RNA	
	KXD1	MT1E			CDC42SE1	
	PSMG3	DYNC2I1			CIC	
	WNT16	ZNF32			TMEM87B	
	UBE2A	LTBP1			SKIV2L	
	HNRNPA1P53	CLPTM1			PATJ	
	ZNF445	SYMPK			ATAT1	
	HPRT1	FST			MARK4	
	MIR8075	RHEBP2			LINC00342	
	MIF	KANSL3			ZSWIM8	
	PSMB3	EXOC7			LINC02762	
	PARP11	ZIC2			LRATD1	
	PECR	LINC00997			PHIP	
	ZNF562	ZC3H15			TOM1L2	
	LSM11	HMGCS1			GGA2	
	UNC119	ARHGAP30			ESRRG	
	KIAA0586	CLTC			NSFL1C	
	MDM2	CFAP70			MCOLN2	
	RPS6	TTC39C			HMGA1	
	TMEM184C	ICAM3			ATP8B2	
	NAA35	CAPZA2			WASHC4	
	ATG4B	ESYT1			ZNF518A	
	PHPT1	NAA30			HELLS	
	ARNT2	SOS2			IMPACT	
	SMIM14	CLDN3			SLC16A14	
	RASSF1	POLR3E			ANKRD36C	

	ETV4	TNRC6A			KATNA1	
	FOSB	CRB3			GJB6	
	HIPK3	ANKRD6			CNOT6	
	PSMB8-AS1	SLC6A17			SGK1	
	GGNBP2	ANAPC2			TRIM39	
	LEMD3	DYNC1I1			BEST1	
	DOCK9	DNAJB14			NUCKS1	
	GAS2L3	IGFBP7				
	CTC1	CASQ1				
	UXT	HSPA14				
	ZNF770	FOXF2-DT				
	SSNA1	ARHGAP10				
	CALM2	NFX1				
	ALKBH4	NOP14				
	FUT10	THAP5				
	HSF1	GPR158				
	AQP11	TCF20				
	GLTP	ATXN7L3				
	HERC6	CDC42BPB				
	SLC38A9	CBS				
	GRAMD1A	EIF4G1				
	UBA6	ALDOA				
	H4C14	FOXN2				
	GGA1	ACO1				
	SIRT6	HOXC12				
	UBE2D2	NUP62				
	RPS26	SELENOT				
	DNMT1	LINC01152				
	TMEM109	PLEKHF2				
	WWOX	ZNF138				

	LRIG2	AGBL2				
	HSP90AA1	SEC23IP				
	SLC5A6	CCDC74BP1				
	ERN1	MAP2K3				
	NECAP1	NCOA4				
	TTLL4	RBM23				
	RYR1	KRT222				
	PGAP6	MAP2K7				
	TMA16	IPO11				
	RICTOR	EEF1B2				
	LOX	WASH6P				
	OST4	OTUD6B-AS1				
	LINC02562	NECAB3				
	GCN1	MLLT1				
	CLU	CST6				
	PSMD6	NUMB				
	ADAMTS6	ARPC5L				
	ZNF771	MYOSLID				
	IL33	ANKLE2				
	CEACAMP10	CCDC14				
	RAB3GAP2	SLC38A4				
	TLN2	DYNC1I2				
	FKBP5	NUS1				
	PI4KAP1	POLM				
	MTMR2	PLAUR				
	CHRNB1	ZFPM2-AS1				
	OR6D1P	ILVBL				
	C14orf93	CCDC136				
	BGN	NALCN				
	TXNL4A	RANBP10				

	EOLA1	UPK2				
	TAF12	CGNL1				
	POLR2F	WDR25				
	HELZ2	CLP1				
	TRAPPC1	LINC00641				
	ACTN4	SLC35A3				
	MTND6P4	TMEM72-AS1				
	CNKSR3	E2F4				
	HPS3	ETV6				
	PMM1	ZNF707				
	ILK	KREMEN2				
	SGTB	EXOC6B				
	HM13	FEM1C				
	INTS2	SRSF10				
	DUSP5-DT	DCTPP1				
	H3C1	FLRT2				
	COLQ	KCTD15				
	RNF181	TFRC				
	RPS8	UPF1				
	ALG10	VMA21				
	GTF2F2	C20orf96				
	MMP10	CNN2				
	LINC00243	OVOL1				
	AP5Z1	NSRP1				
	B3GNTL1	SIK3				
	PPIP5K2	AXL				
	WDR6	FRMD3				
	YIPF1	MTHFSD				
	FANCB	PROC				
	LINC02877	IFT172				

	TRAPPC12	MOB4				
	TMEM256	PLOD1				
	LAMTOR3	DISP2				
	WARS2-AS1	PIGG				
	BICD1	ACOT1				
	TMEM243	PRPF38B				
	FAN1	DGKE				
	ITGAL	CITED2				
	EXOSC4	URI1				
	KRT34	OR2A7				
	SNRNP200	SIKE1				
	APOBEC3A	PHF3				
	FAM120C	BTD				
	TNRC6B	STAMBPL1				
	CLDN22	DNAJC18				
	C9orf85	CLDN4				
	DPH2	KCNK15-AS1				
	MAST4-AS1	DDX50				
	TMED8	ADGRF5				
	OFD1	FBXO5				
	AKAP11	DYNLL2				
	C10orf95-AS1	RSBN1				
	PLCXD2	ZRANB2				
	PTGES	AGGF1P2				
	TMCO1	PLPP1				
	ITPR2	VANGL1				
	CD93	TMEM62				
	TGFBRAP1	BRF1				
	GET3	WDR47				
	TXNDC9	MGAT5B				

	COMMD2	ZFAND4				
	RPL7P49	ZNF544				
	WDR91	MAGEA6				
	MBIP	LINC01389				
	NEMP1	CYP7B1				
	ATP2C2	KRT23				
	SELL	SCLT1				
	DDX41	DDHD1				
	PDZD11	PTPN21				
	XRCC5	TDO2				
	RNPEPL1	HSPA12A				
	RPL19	DPH6-DT				
	TCOF1	NEURL1B				
	USO1	ITPRID2				
	CCDC125	RAB27A				
	KIF3B	WNT10A				
	CFAP221	ASB2				
	CSAD	HABP4				
	NFAT5	CARD8-AS1				
	TOP2B	PILRB				
	LITAF	GRHL1				
	MIR2117HG	ATP1A1-AS1				
	SLC24A2	ZFHX2				
	FARSB	CCT7				
	LINC01503	ZNF107				
	PIK3R4	ATF5				
	FAT4	UHRF1				
	MAML3	RAB12				
	NHLRC2	HMGB1P1				
	DDAH1	DNAJC16				

	AKAP9	CASKIN2				
	GGT7	SRM				
	RPL21	ZKSCAN2				
	ATP6AP2	PGAP2				
	MOCOS	GFUS				
	DSE	PRR15L				
	C18orf54	FSD1				
	SCGB2B2	WNT3				
	TSEN34	LRP11				
	NMRAL1	TMEM117				
	SLC25A47P1	ANGPTL4				
	LRRC73	C2CD2				
	SNORD17	ENGASE				
	RDH10	PNPLA3				
	C1orf112	B3GNT2				
	NECTIN4	ANO1-AS1				
	PLEKHA5	DUSP5P1				
	MSANTD3	FAR2P1				
	CD52	MIOS				
	RNF207-AS1	FZD4				
	POLD4	YARS1				
	FLJ31356	OSER1-DT				
	SLMAP	ATP2A2				
	NLK	CPEB3				
	MED30	FAM241A				
	LINC02606	ZNF33A				
	LINC02747	FAM168B				
	ALG6	MAT1A				
	RPL24	FER				
	HAUS2	CDK16				

	SLC3A2	RAB11FIP2				
	TRPT1	FKBP9				
	CSGALNACT1	OGFRP1				
	EMC6	HSPA6				
	ATF7IP	PIK3C2B				
	SRI	NAPG				
	SART3	ZNF497				
	POC5	PANK3				
	EID2	DAB2				
	DGKD	PI4K2A				
	PRB3	LINC01315				
	ENTPD1-AS1	ADSS1				
	XRN1	ZFP92				
	MARCHF6	TMED10P2				
	IGF2BP3	C3orf80				
	CFAP157	CAPN15				
	LINC01416	RAPGEFL1				
	NF1	USF1				
	CNDP2	CBX7				
	ANKRD10-IT1	RAB20				
	FNBP4	MAP2K5				
	ZNF41	ZBTB40				
	LINC01990	MYO10				
	TNNC2	POGLUT2				
	LINC00707	LINC01116				
	POT1-AS1	TAS2R4				
	TBCA	ZSCAN16-AS1				
	TMA7	SCAF8				
	ADAMTSL1	SNHG4				
	TBC1D2B	LRRIQ1				

	SGO2	ACVR2A				
	IQCK	HOXC13				
	FAM3B	SGCB				
	ARFIP2	NFKB1				
	MAP3K4-AS1	ANKRD18B				
	C1RL-AS1	MFSD5				
	DNPH1	TCHH				
	FYCO1	TRIM28				
	SYF2	DDX6				
	KLF3	PI3				
	KIF20A	YPEL1				
	LIG1	SMPDL3A				
	SPEG	RPP25				
	OSBPL8	ILRUN-AS1				
	CAVIN3	MR1				
	MED7	CCNI				
	SMYD4	TAOK2				
	PPIAP72	ERP27				
	XYLB	PACSIN3				
	ZMIZ2	TUSC3				
	LINC00630	DPF2				
	PDCL3	LEMD1				
	SUCO	KAT2A				
	TERT	TGIF1				
	CAMSAP1	LINC01269				
	SNRPA	PHYHIP				
	H2AJ	TENT5B				
	ANKRD54	FBXO31				
	NRDE2	UGT1A12P				
	TMEM161B-	THBS3				

	AS1					
	NT5C	CASP1P2				
	DVL2	USP32				
	DSG3	ECPAS				
	LMBRD2	LYN				
	MFSD3	SDCBP2-AS1				
	DNM1P51	LRRC49				
	ZC3H7B	IPO5P1				
	MIR3936HG	HNRNPM				
	CNTNAP3B	AGO3				
	PSMA4	ATP6V0A1				
	ASAP1	C22orf39				
	XAF1	GPR155				
	EVI2B	PCNX2				
	HOXA5	PIK3R1				
	FAM183A	SMARCA4				
	PDE4DIPP2	MYO5A				
	PELI1	RAB8A				
	ZBP1	POTEE				
	HACD3	NIFK				
	UOX	STIM2				
	FTH1P16	ZNF638				
	C19orf25	MICA				
	EOLA2	FADS6				
	PMS2P1	PORCN				
	CCNQ	ZNF354B				
	ABCC6P2	ZNF586				
	MIP	ANKRD20A18P				
	SSH2	ARHGAP39				
	PNRC2	ZNF180				

	RERE-AS1	NFKBID				
	SMARCA2	CHUK				
	OAZ2	NUDT16				
	CLCN4	TBKBP1				
	FUT8-AS1	FCHO2				
	LMF2	SDCBP2				
	PPP1R13L	TMEM200A				
	B4GALNT2	CYS1				
	PPIAP46	MED25				
	RNASEH2B	MAN1A2				
	C11orf72	DROSHA				
	NT5C3A	ABHD5				
	ZNF337	TGFBR2				
	TRANK1	KLHL21				
	WDR7	WDR54				
	CKAP4	NPEPPSP1				
	RPAP1	UBXN11				
	TMEM238	SOAT1				
	KIF18B	ABCA5				
	WWP1	CCDC9B				
	HYKK	TNFAIP8L1				
	KLHL23	TPX2				
	FLYWCH1	CAPN5				
	SEC13	TXNDC5				
	FKBP9P1	REEP6				
	TMEM158	WFDC10B				
	PCNX1	METTL22				
	TBCCD1	SNRPD3				
	JKAMP	SNAPC3				
	LINC00184	GABPB1-IT1				

	MIER2	NLRP1				
	CFAP53	KLHDC9				
	STPG1	CFAP251				
	NUDT15	PTGS1				
	TMEM131L	PANX1				
	PHC3	FBXO28				
	LASP1	ARPC1B				
	ERVFRD-3	GLA				
	FADS3	TRIM37				
	HSBP1	ZDHHC16				
	CCDC138	DHRS11				
	TEAD1	NDFIP2				
	KIDINS220	PTPN3				
	RPS15	MEA1				
	KIAA1841	PTGIS				
	TBL1XR1	RASAL1				
	EIF6	RTL8A				
	RBMS1	RDX				
	MIR4664	LRRC28				
	RALA	PABPN1				
	KRT32	PSRC1				
	HERC2	ELOVL5				
	URB2	FKBP4				
	RBM28	PPP1R3E				
	S100A10	SH2D3C				
	ZNF326	RNF130				
	PLA2G6	IL21R				
	PSME4	PTOV1-AS2				
	TRIM69	RPL23AP53				
	HECTD1	NPL				

	POLR2J	LPXN				
	TTYH2	HSD17B1-AS1				
		SYT1				
		ARL4C				
		SUN2				
		IP6K1				
		FAM169A				
		HOXB1				
		KDM6A				
		GABRE				
		AQP7P1				
		PVT1				
		CEP104				
		FAM117A				
		EGLN3				
		ABCG1				
		MCM10				
		FOSL1				
		PLA2R1				
		ZNF772				
		KRT6C				
		RTKN				
		EIF3G				
		KCTD5				
		VWA1				
		ASF1B				
		HEATR3				
		UNK				
		ADGRG1				
		ERI2				

		RPL28				
		METTL2B				
		FZD6				
		ANO9				
		MAMDC4				
		CNTN6				
		FJX1				
		PSMB8				
		KDM2B				
		TLK1				
		RPP25L				
		COG6				
		KCTD18				
		H2BC15				
		PPP2CB				
		B4GALT2				
		C5orf66-AS1				
		APCDD1				
		USP31				
		DCUN1D1				
		IQCG				
		ZMPSTE24				
		CABLES1				
		EPOP				
		ENTPD3				
		CCNT2-AS1				
		NINJ2				
		FAM66C				
		CSGALNACT2-DT				
		HMGN2				

		SNX6				
		FAM117B				
		RTN4				
		C1QTNF1				
		SUPT6H				
		KCMF1				
		HASPIN				
		RAD51B				
		LINC02635				
		TCTN1				
		PPRC1				
		CLN5				
		TRMT10A				
		BMP2K				
		PHC2				
		SON				
		ZNF268				
		RNF10				
		DUSP12				
		CKMT2-AS1				
		LINC02035				
		ENKD1				
		DBNDD2				
		RAB43				
		SOCS2				
		C12orf57				
		FTLP3				
		ADCY9				
		NBEAL2				
		WDTC1				

		MYBL2				
		YPEL4				
		PLPP3				
		DIP2A				
		KCTD6				
		ZNF749				
		DYM				
		ICAM5				
		BMS1P2				
		ORAI2				
		ANKRD26				
		ZNF529-AS1				
		ID4				
		CD302				
		FAF2				
		LCMT2				
		WFIKKN1				
		AGPS				
		MGRN1				
		RAB11A				
		FAM131B				
		EEA1				
		DLX2				
		PPP3R1				
		GPX2				
		MUC4				
		HAP1				
		RNF217				
		POGLUT3				
		IRF6				

		PPP1R21				
		GNA11				
		TRAM1L1				
		OR2A1-AS1				
		GCC1				
		B4GAT1				
		RNF216				
		SP100				
		CDHR3				
		SUSD3				
		SFT2D1				
		TTLL10				
		RAPGEF6				
		GTDC1				
		FIBP				
		TEDC1				
		SMC3				
		VCL				
		SEC62				
		LINC01882				
		NHSL1				
		AURKA				
		DBP				
		IFT52				
		RAB30				
		ITGB8				
		DNAAF1				
		PLGLB1				
		PDPK1				
		N4BP1				

		C20orf197				
		RHOU				
		TP53I13				
		CFAP69				
		SNX21				
		LINC00944				
		IFT140				
		ARHGEF34P				
		ABCA1				
		CHAF1A				
		SPSB3				
		TRAK1				
		SLC45A4				
		RUFY2				
		KIAA1549				
		SYT11				
		LRRC14				
		CTSO				
		ZCCHC8				
		TSPYL2				
		ZC2HC1C				
		RPL32				
		RAB11B				
		HMGB2				
		TMOD3				
		PNISR				
		WDR5				
		PKDCC				
		CUL9				
		TMPRSS11A				

		SNX1				
		GIHCG				
		UBE2W				
		ZSCAN9				
		ARHGAP25				
		AAR2				
		LHPP				
		VAV2				
		SNORA33				
		LINC02551				
		MARF1				
		EDC3				
		MAFG-DT				
		FAIM				
		DTWD2				
		PPP1R3C				
		ARHGEF35-AS1				
		OR2A20P				
		PRC1				
		PBX1				
		LINC01451				
		GRB10				
		TVP23B				
		VKORC1				
		SULT1A2				
		MADD				
		POLR1F				
		ITPA				
		LRRC37B				
		HOMER1				

		MIA2				
		TEX21P				
		TES				
		RHNO1				
		ATP13A1				
		GAREM2				
		ZBTB42				
		ZNF611				
		ZNF699				
		CARNMT1				
		NXNL2				
		EVC				
		CDC42EP5				
		CP				
		ABCC2				
		C5orf15				
		RPS17				
		PEDS1				
		VSIG1				
		GABARAP				
		TMEM167B-DT				
		SH3BP5L				
		CACNB3				
		TMEM107				
		TATDN1				
		TOX2				
		LY6G5B				
		B4GALNT3				
		ZNF654				
		C11orf1				

		LINC00653				
		LIX1L				
		EIF2S1				
		TWF2				
		SH2D2A				
		KIAA1191				
		PRR14L				
		KMT2B				
		RN7SL541P				
		LINC02005				
		CCL22				
		C12orf54				
		RPS18P9				
		DCAF15				
		SEPTIN2				
		MARCHF9				
		SNX18P7				
		SLC2A13				
		RHBDD3				
		MYLK				
		EPS8L1				
		ZDHHC2				
		LENG8				
		SPAG17				
		CLDN9				
		SPAG9				
		HSD17B11				
		ROCK1				
		ACE2				
		EMILIN2				

		ZBED1				
		TBC1D10A				
		PTPA				
		PLXNB3				
		GCLC				
		UIMC1				
		TRPC4AP				
		GNPDA1				
		LINC02767				
		CCAR1				
		ACSL4				
		TBCD				
		ZNF665				
		TNPO2				
		KMT2C				
		PACC1				
		RAET1E-AS1				
		TACC3				
		ZSCAN23				
		HRH1				
		TMEM9B				
		PLA2G4F				
		TMEM14B				
		GPR1				
		MAP3K2-DT				
		TNPO1				
		CMBL				
		C2				
		GTF2E2				
		PRIM2				

		KIAA0825				
		KRT40				
		ZNF239				
		NEK11				
		CDADC1				
		MT-TE				
		ZNF436				
		ATXN7L1				
		TTC21A				
		SAPCD1				
		UBAP2				
		TNIP1				
		DBF4B				
		ZNF354A				
		MTMR6				
		RNF38				
		SOX2				
		C12orf29				
		CIRBP				
		SUGP2				
		P2RX4				
		XRCC3				
		FABP6				
		IL36RN				
		NF1P1				
		TCAM1P				
		HSD11B2				
		PRPF8				
		EN1				
		HEATR4				

		MYH9				
		SOAT2				
		MPHOSPH8				
		SOX7				
		SEC31B				
		SEMA4B				
		GPR108				
		HENMT1				
		LPIN3				
		DUSP2				
		FCHO2-DT				
		CHP1				
		CCDC74A				
		RFLNB				
		TMUB2				
		PEX11G				
		ZNF56				
		LONP2				
		SNHG1				
		RAB5A				
		PIMREG				
		MAPKAPK3				
		INKA2				
		ITGB7				
		TMOD2				
		U2AF1L4				
		DANCR				
		POLR1B				
		TBX3				
		ZNF639				

		CEP20				
		SPTB				
		PGLS				
		SLC16A8				
		TM2D1				
		LURAP1L				
		MIR1282				
		P2RX6				
		NAT10				
		ANKRD13C				
		MIR4666A				
		COL8A2				
		SRP72				
		TESK1				
		TMX3				
		DNAJC24				
		PIH1D1				
		RPN2				
		PRTFDC1				
		LIN7C				
		RASSF10				
		ZFPL1				
		LLGL2				
		STRN4				
		APOBR				
		PTER				
		NUP85				
		TTC30B				
		TARBP1				
		AKAP3				

		INTS13				
		PTPN4				
		ZCCHC24				
		RRP15				
		INHBA				
		FUZ				
		RPL23				
		MAGT1				
		CCNT1				
		MRGPRX3				
		PARP12				
		MED27				
		STRCP1				
		MYNN				
		ZC3H4				
		CTSC				
		MT1F				
		ZNF439				
		BZW1				
		HEY2				
		XPO1				
		BAHD1				
		LCN12				
		SLC35C2				
		SET				
		MT-TA				
		APLF				
		SPATA13				
		C2orf49-DT				
		PIAS3				

		GIPR				
		ERN2				
		PPHLN1				
		RALGAPB				
		STXBP4				
		GIT1				
		EXOC4				
		MZF1				
		DUOX1				
		PLAGL2				
		WDR76				
		FBXO44				
		KLHDC7B				
		TIMP4				
		KANSL2				
		MTA3				
		SF1				
		SNORD104				
		SZRD1				
		VPS37D				
		TOLLIP-AS1				
		ENDOU				
		DPEP1				
		OSBP				
		TRIP11				
		ARSK				
		ZNF668				
		GAK				
		CPNE5				
		ARHGAP33				

		LINC02178				
		ARHGEF6				
		MANEA-DT				
		HOXB5				
		SLC52A1				
		TBC1D13				
		ZNF134				
		HACL1				
		RAD23B				
		ABTB1				
		FAM200B				
		APCDD1L				
		ILF3-DT				
		USP6NL				
		LINC01572				
		LCLAT1				
		NUP42				
		PGAM1				
		DHX8				
		WDR93				
		DENND2D				
		CINP				
		UBE2O				
		TMED5				
		AQP3				
		LINC02569				
		SCNN1B				
		LCOR				
		LIN52				
		SP140				

		MLXIPL				
		LINC02580				
		SUFU				
		LUC7L3				
		LINC01010				
		P2RY6				
		NAA20				
		UBE2D1				
		C21orf91				
		NUDT17				
		ARFRP1				
		SLC13A4				
		CRYZL1				
		FGFR1OP2				
		KHSRP				
		PITPNA				
		NUTF2				
		C15orf62				
		YJU2				
		CUEDC2				
		ZNF192P1				
		STARD3NL				
		TADA2B				
		BBS7				
		STK40				
		TMEM208				
		CRTAP				
		MARCHF7				
		NSD2				
		BNIP2				

		CDC20				
		MCOLN3				
		RYBP				
		MINDY3				
		SH3BP1				
		FBXO42				
		DNAH14				
		TFEB				
		GRHL2				
		TBK1				
		PCGF2				
		ZNF594				
		FAM43A				
		TMEM217				
		MCF2L				
		TAF6				
		SNAP25-AS1				
		MICE				
		TMEM106A				
		BCAM				
		THEMIS2				
		RXRA				
		CHD9				
		ZNF496				
		SLC7A11				
		INTS8				
		ARL6				
		TNKS2				
		SAE1				
		RPL27				

		WIZ				
		RUVBL2				
		GTF2H3				
		TOB2P1				
		ATXN7L2				
		DOLPP1				
		TRAPPC2L				
		LPP-AS2				
		BTRC				
		MIR100HG				
		CSRNP1				
		HAUS4				
		SUPT3H				
		GBF1				
		MCRS1				
		OOSP3				
		CENPBD1P1				
		FSIP2				
		LINC02084				
		BUB3				
		PECAM1				
		PHLDB2				
		ARHGAP21				
		RIC8B				
		INPP5E				
		SENP2				
		PTPN11				
		GRHL3				
		TRIO				
		APOC1				

		ARF3				
		ARMC9				
		ANKRD50				
		NOL11				
		ARHGAP31				
		PLXNA2				
		ERGIC1				
		MARVELD2				
		MYO15A				
		LINC02519				
		MIR137HG				
		BET1				
		CHTOP				
		PRMT3				
		C3orf38				
		PITX2				
		JAK3				
		AGAP1				
		TENM3				
		GRASLND				
		SAMD1				
		PRAG1				
		FAM161B				
		CYP2A13				
		MRAS				
		ORC5				
		ZC4H2				
		THUMPD2				
		RAB35				
		SUSD1				

		TRMT11				
		MAP1S				
		LTA4H				
		C12orf56				
		CD44				
		ZNF780B				
		RNF4				
		PSMC6				
		PLEK2				
		ENY2				
		FAM83D				
		CDPF1				
		CD83				
		SLC35A1				
		TBC1D23				
		KLF5				
		LINC02701				
		TP53BP1				
		CWC25				
		BANP				
		LAMA3				
		C1orf43				
		ABALON				
		GSS				
		ARPP19				
		MAGED2				
		LINC02323				
		TAGLN				
		VASH1				
		KIF5B				

		USP3-AS1				
		CLMP				
		TMEM44				
		APBA3				
		PRPF40B				
		DMAP1				
		TRMT61A				
		VAPA				
		ABHD8				
		BICD2				
		ATL1				
		PM20D1				
		CCDC190				
		LINC00514				
		PDE6A				
		TIAM2				
		MED16				
		SART1				
		PTBP2				
		CD58				
		YWHAZ				
		NACC2				
		UTP4				
		PLSCR4				
		H4-16				
		CENPC				
		SLC44A3-AS1				
		TENM1				
		ZNF696				
		CCN6				

		ACTR5				
		MT1L				
		RPS2				
		RGP1				
		SEC22A				
		LINC01410				
		DM1-AS				
		SMCO4				
		TRMT10B				
		RPL37				
		TLE2				
		PUDP				
		C1orf21				
		UBE2T				
		NLRC5				
		ZBTB24				
		CRACR2B				
		SPATA2				
		ZCCHC14				
		UBTD2				
		NOP14-AS1				
		WTAPP1				
		SOD2-OT1				
		MED4				
		GJC1				
		LINC02878				
		ELAVL1				
		PRKAA1				
		CD2AP-DT				
		SCD				

		ASB9				
		CCNG2				
		MFSD14C				
		TRAM2				
		SRCIN1				
		SNUPN				
		GLI1				
		TMTC4				
		MMP19				
		PAFAH1B3				
		RAB10				
		PPM1N				
		WWC2				
		GOLGA6L5P				
		LNCTAM34A				
		STAT5A				
		YY1				
		TMEM187				
		GOPC				
		DZIP1L				
		RNF224				
		GBP1				
		TCEAL9				
		PRPF39				
		IFI6				
		MFSD4B				
		P2RX7				
		DST				
		PNO1				
		NAGK				

		SMIM31				
		SCD5				
		PIPOX				
		FAAHP1				
		GNG12-AS1				
		IGHMBP2				
		CIAO2B				
		SMIM30				
		RAD52				
		TSACC				
		NHEJ1				
		SAMHD1				
		STIP1				
		CCDC22				
		MGAT2				
		KPNA1				
		RRP1B				
		RHOBTB1				
		CCDC106				
		BBS5				
		C4B				
		PLBD2				
		ARRDC3				
		CENPH				
		HMCN2				
		UNC79				
		CNOT6LP1				
		PTK7				
		BLCAP				
		FAM182B				

		HCN3				
		TUB				
		LDLRAD2				
		MEX3C				
		PAPOLA				
		PHF21A				
		HAAO				
		DUSP8P5				
		LINC00638				
		LINC01135				
		RPP14				
		ZNF213				
		ACTR1B				
		EMC9				
		HERC2P3				
		DGKQ				
		KLHDC7A				
		KRT83				
		DCAF6				
		GPR63				
		CA11				
		GAS2L2				
		AASDHPPT				
		TMC8				
		AGA				
		CYP1A1				
		ZFP91				
		LYRM4-AS1				
		ABCA4				
		ADAM21				

		CDC34				
		MFSD9				
		SNORC				
		CAMK4				
		TP53BP2				
		TRPV3				
		IGF2R				
		TSGA10				
		CDCA3				
		PACRGL				
		EXT2				
		LINC01695				
		ODF3B				
		GTF2F1				
		HMGA2				
		C15orf56				
		ZNF500				
		DHX15				
		DDX20				
		IL18				
		MALINC1				
		CIPC				
		ENOX2				
		SNHG19				
		ATIC				
		ZNF22-AS1				
		NMT2				
		HSD17B1				
		MAP6D1				
		SHLD2				

		ZSCAN2				
		PUM1				
		WDR5-DT				
		TRIM13				
		ABHD2				
		NEB				
		ENDOV				
		TK1				
		RFX1				
		CECR7				
		TMEM267				
		PCNT				
		TTLL1				
		ABHD17AP4				
		C2orf68				
		NUPR2				
		ESD				
		POC1B-AS1				
		CNFN				
		WDR5B				
		UBE2K				
		PGGT1B				
		RTF1				
		SEPTIN10				
		SLC31A1				
		CDV3				
		TPP1				
		TDG				
		UBE2SP1				
		ZNF815P				

		NOC4L				
		CENPA				
		AMIGO1				
		XKR6				
		UBE2R2				
		ARMC2				
		ZNF252P-AS1				
		POLL				
		AHNAK2				
		TFIP11-DT				
		RRP1				
		CCDC113				
		LINC00865				
		KCNMB2-AS1				
		SCAF1				
		GALNT13				
		CHD3				
		SEPTIN3				
		HLA-DRB9				
		RPS4XP16				
		YBX1P2				
		WNT7A				
		LINC00339				
		PDXDC1				
		RPRD1B				
		MIF4GD-DT				
		KCTD8				
		POMK				
		TOE1				
		TOB1				

		RCL1				
		ZNF346-IT1				
		PHLPP2				
		NAGPA				
		PPCDC				
		VWA7				
		CCDC157				
		ZNF230				
		KATNIP				
		LRRC15				
		CD36				
		CCDC96				
		TMEM140				
		VPS25				
		CLK4				
		CDIP1				
		PAGR1				
		NIP7				
		ZDHHC9				
		LINC01232				
		PRRT2				
		FAM172A				
		FOLH1				
		HPCAL4				
		MC1R				
		CD2AP				
		RNPEP				
		NUDT4				
		IFI44				
		SETD1A				

		TCHP				
		ZNF316				
		FNDC5				
		GPS2P2				
		ALKBH6				
		RPL35AP9				
		RNF170				
		GAB3				
		ACRBP				
		IFT57				
		NR2C2AP				
		POLE3				
		PCDHGA4				
		GSTM2				
		USP12				
		HOXC9				
		FOXN1				
		BCCIP				
		POC1B				
		PLEKHA3				
		IFT80				
		MIER1				
		APLN				
		ATF1				
		EXOSC2				
		ZDHHC8				
		EPSTI1				
		ZSCAN22				
		MACROH2A2				
		PRKAB1				

		SIDT2				
		TCF7L1				
		TYMS				
		ISOC1				
		NMRAL2P				
		CD177				
		FAM234A				
		TRABD2B				
		N4BP2L2-IT2				
		COPS2				
		PSMA5				
		MGST2				
		LRP5				
		FSIP2-AS1				
		RPAIN				
		RSPO4				
		DPM1				
		ZFP2				
		ZNF726				
		CXCL8				
		PTP4A3				
		SVIL2P				
		RIN2				
		HNRNPDL				
		ZSCAN5A-AS1				
		IGFBP7-AS1				
		RAVER1				
		PLK4				
		CGB5				
		BCL3				

		SMNDC1				
		ZNF175				
		ATP6V0B				
		ZNF561				
		GORASP1				
		URM1				
		UPF3A				
		RASSF7				
		FAM167B				
		C1QTNF2				
		ATP6V1C1				
		SLC15A3				
		EVA1C				
		SLC2A3P2				
		AUP1				
		DNASE1				
		TMEM115				
		PKMYT1				
		VAV1				
		SKAP1				
		ADK				
		MYH11				
		LINC01138				
		ARAP1				
		PAQR3				
		ZBED4				
		PIGZ				
		ASS1P2				
		KLHL41				
		CARD17				

		RPL34				
		BRF2				
		CFAP94				
		TYRO3				
		ORAI1				
		THOC7				
		FAM160A2				
		SLC35F6				
		NINJ2-AS1				
		RECQL				
		MATN1-AS1				
		CDON				
		ARHGAP27				
		PABPC3				
		HAUS3				
		CTAGE4				
		AK7				
		ALMS1-IT1				
		PSD2				
		COX5BP6				
		EXOSC8				
		GRK3				
		HNRNPA0				
		MYL5				
		RSBN1L				
		IBSP				
		ZNF317				
		ENTPD4				
		STXBP5				
		MINDY1				

		NUAK2				
		HIPK2				
		VIRMA				
		GIMAP6				
		LINC00667				
		SNORA73B				
		AP4B1				
		KLHDC7B-DT				
		AMZ2				
		MAPKAPK5				
		RIPOR1				
		UPF3B				
		RAPGEF1				
		NR2C1				
		LINC00910				
		RSPH4A				
		UBALD2				
		POLR3K				
		LINC02615				
		FRG1EP				
		ACOT8				
		FES				
		GTF2A1				
		CANT1				
		ZNF516				
		TMEM102				
		PHETA1				
		MAP7D3				
		PPARGC1A				
		ZNF782				

		EGFL6				
		PRDM2				
		EXD3				
		POLR1G				
		MYO7B				
		SMIM7				
		C15orf65				
		G3BP2				
		MAP3K14				
		KLF2				
		ARHGAP32				
		MLLT10				
		HOXD13				
		TSG101				
		CYTH1				
		TTPAL				
		TONSL-AS1				
		RPS20P14				
		DEGS2				
		ZNF674				
		RPS6P25				
		CCDC17				
		MFHAS1				
		ANKDD1B				
		SPINT1				
		VWDE				
		IFT122				
		ODAM				
		FRMD4A				
		TSNAX				

		SARNP				
		CTC-338M12.4				
		MATN1				
		NFIB				
		HMGB1				
		RUNDC1				
		C1orf35				
		TTLL9				
		TANGO2				
		LINC02031				
		TYRP1				
		SLC15A2				
		ZNF543				
		M6PR				
		PCOLCE				
		OTUD6B				
		EPS8L3				
		MUC12-AS1				
		NFYAP1				
		PKNOX1				
		PIGS				
		FUT6				
		C19orf47				
		TSC22D1				
		SOX15				
		P3H3				
		TMEM144				
		ABCA7				
		CHMP4B				
		DHX9				

		PHF7				
		RRBP1				
		RSRC2				
		TMEM260				
		CDH24				
		TGS1				
		RWDD2A				
		NANP				
		PSENEN				
		KDM7A-DT				
		RPL31				
		LARP6				
		GUSBP3				
		BTN2A3P				
		PPM1D				
		THNSL2				
		TNNT3				
		TMEM209				
		SFSWAP				
		HSPA4				
		SYK				
		PIP4P2				
		H2BC18				
		CELSR2				
		UBQLN1				
		NFKBIB				
		SEC61G				
		UMAD1				
		FANK1				
		SFR1				

		RPAP3				
		GTF2IP12				
		ZFAT				
		GLUD1P3				
		PLRG1				
		STAM				
		SYNCRIP				
		XPO5				
		SETBP1				
		RTCA-AS1				
		PDIA3P1				
		C11orf24				
		CHEK1				
		PTPN2				
		RRM1				
		FAM214B				
		EID3				
		SUSD2				
		LINC01355				
		CD24				
		FNDC4				
		TRPM2				
		EP400P1				
		HERC2P4				
		LINC02535				
		CDC5L				
		SLC16A12				
		SOCS7				
		RNF125				
		RGS2				

		CDC42EP4				
		PFDN1				
		UPP1				
		LINC01550				
		TTLL12				
		MISP3				
		TNFRSF11B				
		GANAB				
		EMSLR				
		PCDHB12				
		POTEF				
		EFEMP1				
		PSG5				
		LRIF1				
		FAR2P4				
		STARD5				
		CHM				
		LSM12P1				
		APH1A				
		FBN2				
		GAB1				
		THRA				
		MT-TF				
		NOP58				
		BEND6				
		HIP1				
		ZNF713				
		TIAM1				
		RRS1				
		TNIP2				

		BRD3				
		EAPP				
		ZMYND8				
		RNF5P1				
		IGFBP4				
		GTF2IP1				
		DNPEP				
		ZNF670-ZNF695				
		ATP6V1B1				
		DECR2				
		PDIA5				
		CYP46A1				
		LCAT				
		SP2-AS1				
		BACE1				
		CCDC77				
		PABPC1				
		RPE				
		LINC01569				
		CTSL				
		BAZ1A				
		ZNF460				
		RAD51AP1				
		VPS4B				
		UBE2I				
		ASL				
		LMTK3				
		HIPK1-AS1				
		YBX1				
		CDC20P1				

		ORC4				
		ARRDC2				
		ZNF12				
		CRTC1				
		INTS6-AS1				
		IFT22				
		LINC01127				
		LINC01979				
		SERGEF				
		RAB11FIP5				
		PCDHGA7				
		HGSNAT				
		TRIM62				
		EPM2AIP1				
		CD320				
		DGKZ				
		JAZF1				
		SCML1				
		TAX1BP3				
		PTENP1-AS				
		EIF2AK1				
		PMEL				
		CTDSPL				
		ST3GAL4				
		TFAP2A-AS2				
		SIRT1				
		RABGAP1L-DT				
		UBE2Q2P1				
		SRSF11				
		LINC00702				

		MAGED1				
		DPY19L2P1				
		ETF1				
		ING3				
		CEP131				
		SIAE				
		ALS2				
		SEPTIN7				
		NLRP2				
		SP9				
		BRD9				
		RNF34				
		IRF2BPL				
		USP33				
		DDX23				
		NSMF				
		BLACAT1				
		ZNF24				
		BCYRN1				
		LINC01869				
		SMKR1				
		ANAPC5				
		KRT80				
		PEAR1				
		GABARAPL2				
		PTCHD4				
		COMMD1				
		TMEM135				
		LTV1				
		RHOJ				

		STX19				
		ZRANB3				
		TBC1D2				
		POLR2KP1				
		TEX264				
		KPNB1				
		CEP250				
		LINC01060				
		FAAP24				
		TRIM9				
		ZNF764				
		RABEPK				
		S1PR1				
		ZNF330				
		KRT86				
		GDF9				
		HMGN3-AS1				
		FBXO48				
